# Isoleucine Side Chains as Reporters of Conformational Freedom in Protein Folding Studied by DNP-Enhanced NMR

**DOI:** 10.1021/jacs.5c04159

**Published:** 2025-04-26

**Authors:** Leonardo Levorin, Nina Becker, Boran Uluca-Yazgi, Luis Gardon, Mirko Kraus, Marc Sevenich, Athina Apostolidis, Kai Schmitz, Neomi Rüter, Irina Apanasenko, Dieter Willbold, Wolfgang Hoyer, Philipp Neudecker, Lothar Gremer, Henrike Heise

**Affiliations:** 1 Institute of Physical Biology, Heinrich-Heine-Universität Düsseldorf, Düsseldorf 40225, Germany; 2 Institute of Biological Information Processing (IBI-7: Structural Biochemistry), Forschungszentrum Jülich, Jülich 52425, Germany

## Abstract

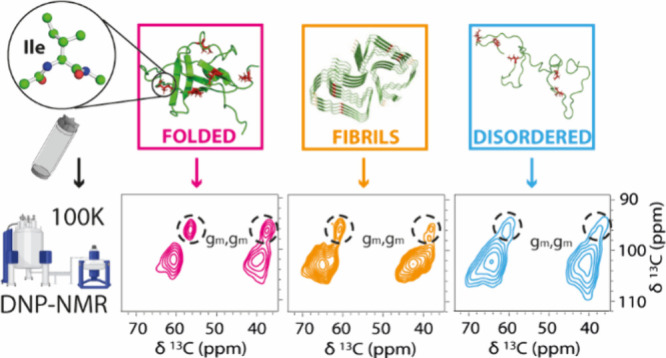

Conformations of protein side chains are closely linked to protein function. DNP-enhanced solid-state NMR (ssNMR), which operates at cryogenic temperatures (<110 K), can be used to freeze-trap protein conformations, including the side chains. In the present study, we employed two-dimensional DNP-enhanced ssNMR to get detailed insights into backbone and side chain conformations of isoleucine. We used different amino acid selectively labeled model proteins for intrinsically disordered proteins (IDPs), denatured and well-folded proteins, and amyloid fibrils. ^13^C chemical shifts are closely correlated with secondary structure elements and χ_1_ and χ_2_ angles in isoleucine side chains. Thus, line shape analysis by integration of representative peak areas in 2D spectra provides an accurate overview of the distribution of backbone and side chain conformations. For the well-folded proteins GABARAP and bovine PI3-kinase (PI3K) SH3 domain, most Ile chemical shifts in frozen solution are well resolved and similar to those observed in solution. However, line widths of individual Ile residues are directly linked to residual mobility, and line broadening or even signal splitting appears for those Ile residues, which are not part of well-defined secondary structure elements. For unfolded PI3K SH3 and the IDP α-synuclein (α-syn), all Ile side chains have full conformational freedom, and as a consequence, inhomogeneous line broadening dominates the cryogenic spectra. Moreover, we demonstrate that conformational ensembles of proteins strongly depend on solvent and buffer conditions. This allowed different unfolded structures for chemical and acidic pH denaturation of the PI3K SH3 domain to be distinguished. In amyloid fibrils of α-syn and PI3K SH3, chemical shifts typical for β-strand like secondary structure dominate the spectra, whereas Ile residues belonging to the fuzzy coat still add the IDP-type line shapes. Hence, DNP-enhanced ssNMR is a useful tool for investigating side chain facilitated protein functions and interactions.

## Introduction

Protein side chain conformations are closely related to protein function since they play a key role in enzyme active site chemistry and interaction with small molecules or other ligands.^1,2^ At physiological temperature in solution, side chains of amino acid residues in certain regions of a protein may still exhibit substantial conformational freedom, which results in rapid averaging of an extensive conformational ensemble in many spectroscopic experiments. In crystal structures, on the other hand, most side chains are confined to one energetic minimum, which does not necessarily represent the full conformational space sampled by a residue. Likewise, NMR structures of proteins in solution are often based on averaged J-couplings, which reflect average values of torsional angles instead of the actual rotamer distribution, which may also distort the representation of side chains in solution NMR structures. Dynamics and flexibility of protein domains can be mapped by residual dipolar couplings and NMR relaxation, and combined with MD simulations to obtain conformational ensembles at ambient temperatures.^3−6^ Recently, several approaches have been developed to experimentally determine conformational ensembles directly in fully or partly disordered proteins in frozen solution^7−12^ or in lyophilized form^13^ by solid-state NMR-spectroscopy, using conformation-dependent chemical shifts^14,15^ as reporters for secondary structure. Upon freezing in a cryoprotectant medium, the exchange between different conformations is halted, and all conformations sampled by each nucleus are present with their respective probability.^7,8,16−19^ Furthermore, the combination of frozen solution NMR with dynamic nuclear polarization (DNP) makes it possible to study distributions of conformations in large proteins with high sensitivity.^9,12,20,21^ After focusing on the backbone conformations in a previous study,^9^ we started to investigate the conformational space sampled by isoleucine side chains. The chemical shifts of isoleucine (Ile) side chain carbons strongly reflect the side chain torsion angles χ_1_ and χ_2_, which each have three energetic minima at ∼60°, ∼180°, and ∼300°, referred to as gauche+ (g_p_), trans (t), and gauche- (g_m_), respectively.

**Figure 1:**
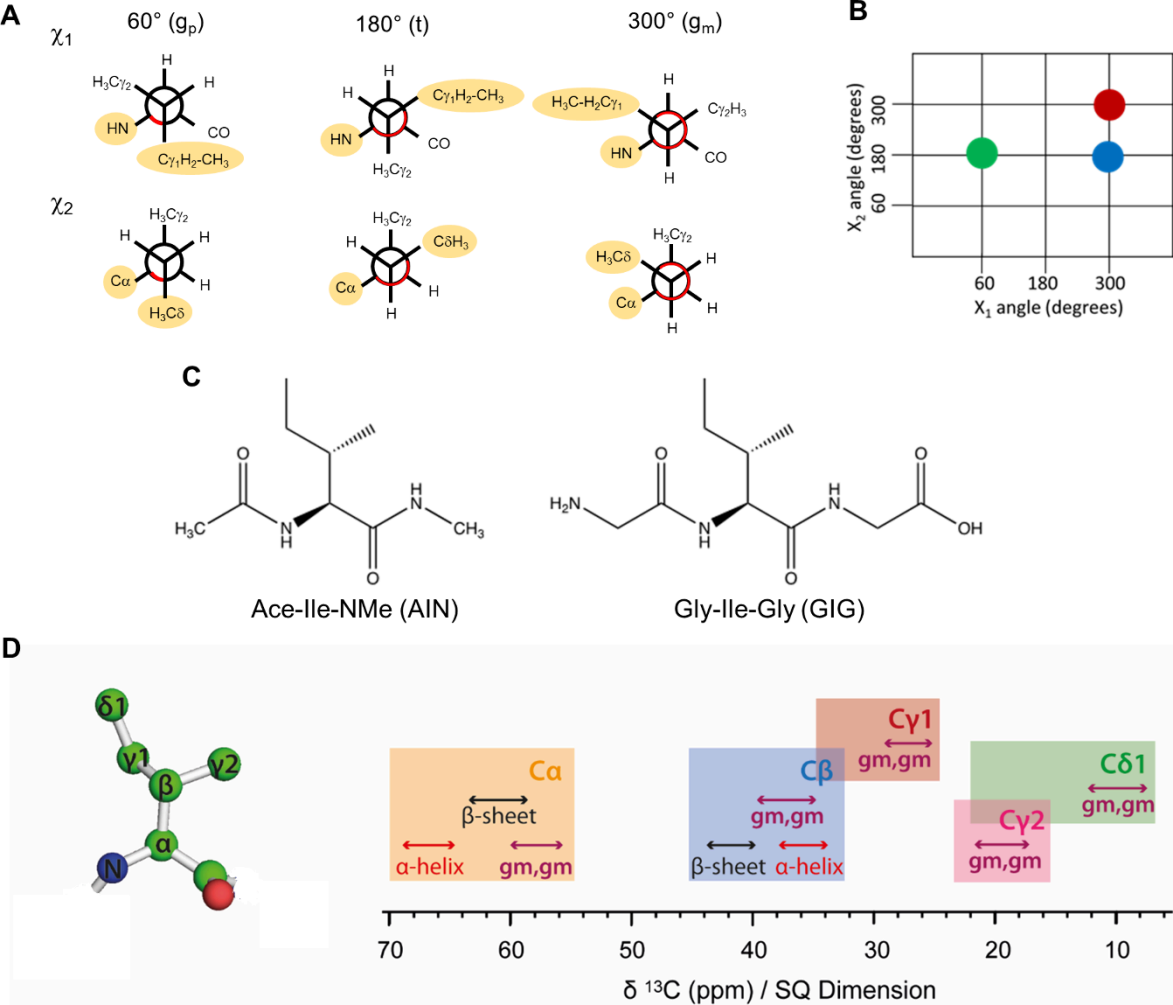
(A) Newman projections of the χ_1_ and χ_2_ dihedral angles for isoleucine. Correlation between χ_1_ and χ_2_ dihedral angles. (B) The most populated three rotameric states (g_m_,t), (g_m_,g_m_), and (g_p_,t) are highlighted with a red, green, and blue circle, respectively (right). (C) Structure of the random coil mimic peptides AIN and GIG. (D) Ile side chain structure (left; heavy atoms only) and typical chemical shift regions for each Ile side chain ^13^C (right).

Density functional theory (DFT) calculations of rotameric states from ^13^C chemical shifts as well as from scalar couplings have shown that three of the nine possible rotameric states. χ_1_ and χ_2_ angles in the combinations (g_m_,t), (g_p_,t), and (g_m_,g_m_), represent over 95% of all rotameric states sampled by the side chains in solution at ambient temperature (Figure 1A,B).^20^

In our previous work, the rotamer distributions of isoleucine in two random coil peptide mimetics, Ace-Ile-NMe (AIN) and Gly-Ile-Gly (GIG) (Figure 1C), were calculated from the experimental chemical shifts and scalar couplings for two peptides. ^13^C chemical shift distributions obtained from DNP-enhanced solid-state NMR spectra of these peptides in frozen solution supported the finding that the random coil sampling of AIN and GIG is significantly different from that obtained from the average of all isoleucine side chains in a large set of high-resolution crystal structures.^20^ In particular, the Cδ1 chemical shift is highly sensitive to the χ2 torsion angle; in the g_m_ conformation the Cδ1 methyl group has two γ-gauche substituents (Figure 1A), which reduces the chemical shift by ∼5.5 ppm.^22^ Typical NMR chemical shift values for Ile ^13^C are shown in Figure 1D.

In this contribution, we adopt the approach to exploit isoleucine residues as structural reporters for backbone and side chain conformational freedom in proteins recombinantly expressed with uniform ^13^C,^15^N isotope labeling exclusively for isoleucine residues.^23^ We studied three different proteins representing well-folded, intrinsically disordered, misfolded and fibrillar states by DNP-enhanced solid-state NMR-spectroscopy in frozen solution, under different conditions.

As an example of a stable, well-folded protein with a defined secondary and tertiary structure, we studied the γ-aminobutyric acid type A receptor-associated protein (GABARAP), a 117 amino acid residue protein playing an important role in autophagy.^24,25^

In contrast, intrinsically disordered proteins (IDPs) can readily adopt different secondary structures under different conditions.^26−29^ One member of the group of IDPs is the so-called ‘protein-chameleon’ α-synuclein (α-syn),^30^ and its aggregation is associated with various synucleinopathies including Parkinson’s Disease, dementia with Lewy bodies and multiple system atrophy.^31^ In its monomeric form α-syn is fully disordered, upon fibrillation the extreme N- and C-termini stay disordered while the middle region adopts a cross β structure forming the fibril core.^32^ Therefore, Ile-labeled α-syn was chosen as a model protein to study a natively unfolded monomeric as well as a fibrillar state.

The bovine PI3-kinase (PI3K) SH3 domain is a well-studied model protein for the investigation of protein folding, unfolding and aggregation.^33^ The structure of natively folded PI3K SH3, a globular domain consisting of 86 amino acids, from bovine PI3K^34^ and its human homologue^35−37^ has been well-characterized by X-ray crystallography and NMR spectroscopy. At low pH, the protein unfolds and forms amyloid fibrils,^33,38,39^ whose structure was characterized by solid-state NMR spectroscopy^40^ and more recently in high resolution by cryoEM.^41^ Here, by using Ile residues as structural reporters, we investigate the different globular, unfolded and fibrillar conformations of PI3K SH3 as a third model system.

## Results

### Rotational Freedom in a Folded Protein: GABARAP

**Figure 2:**
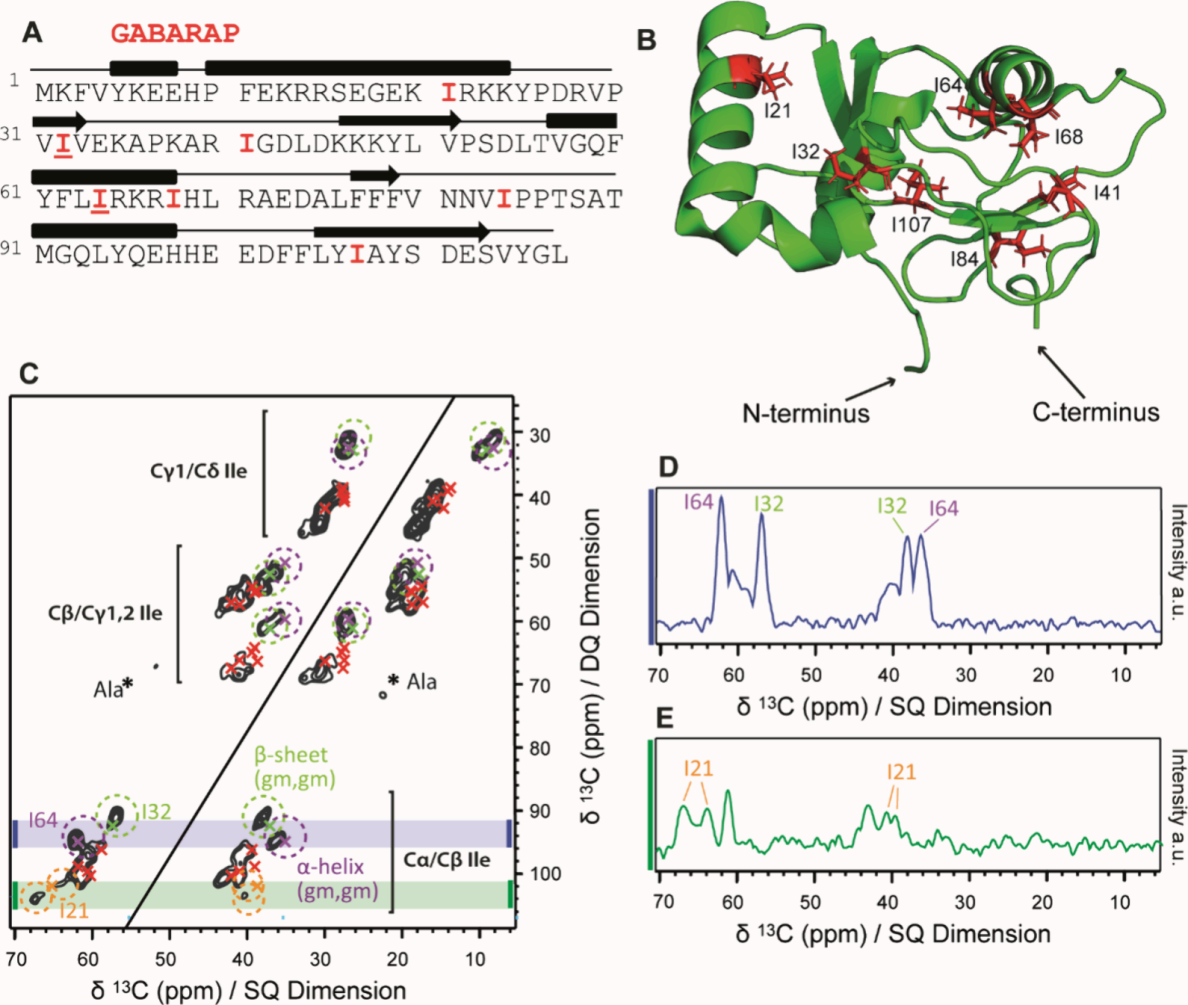
(A) Primary sequence of GABARAP with the positions of isoleucine residues highlighted in red. The secondary structure elements α-helix and β-strand are depicted in rectangles and arrows, respectively. (B) 3D structure of GABARAP with Ile residues labeled and marked in red (1KOT).^42^ (C) 2D ^13^C–^13^C correlation DQ/SQ spectrum of GABARAP recorded at cryogenic temperatures in frozen solution. Green and purple circles show typical regions for β-strand and α-helical (g_m_,g_m_) states, respectively; green (I32), purple (I64), orange (I21), and red (all other Ile residues) crosses mark the experimental chemical shifts from solution NMR.^43^ The splitting of the I21 Cα–Cβ correlation in frozen solution is highlighted using orange circles. Ala cross peaks are marked with asterisks. The spectrum was Fourier transformed without apodization of the FIDs in both dimensions. (D, E) Significant 1D projections extracted from the marked areas of the original 2D data set shaded in gray in panel (B).

The globular protein GABARAP was selected as a candidate for studying residual dynamics in a well-folded protein by comparing solution NMR data measured at room temperature to DNP-enhanced data recorded in frozen solution at 100 K. As the seven isoleucine residues are distributed all over the sequence, they represent all parts of the protein (Figure 2A,B). Three isoleucine residues are located in α-helices (I21, I64, and I68), two in β-strands (I32 and I107), and two in loop regions (I41 and I84) of the protein.

We recorded a 2D double quantum/single quantum (DQ/SQ) spectrum for selectively Ala- and Ile-labeled GABARAP in frozen solution (Figure 2C). DQ/SQ experiments were preferred over standard proton driven spin diffusion spectra (PDSD, Figure S1B), since they show only one bond correlations and, therefore, allow a full discrimination between Cγ2 and Cδ1 resonances, which partially overlap in PDSD spectra due to their similar shift range. In this folded protein, the ^13^C resonances are well-dispersed at ambient and cryogenic temperatures, and a good resolution with ^13^C line widths below 1.5 ppm for most resonances was obtained even at 100 K. Ala cross peaks, which are outside of the relevant spectral area for Ile cross peaks, are relatively weak, possibly due to isotopic scrambling, and are not further evaluated in this study.

As shown in Figure 2C, most of the well-separated DNP-NMR peaks in frozen solution match the solution NMR chemical shift values, allowing the identification of the spin systems for all seven isoleucine residues (Figure S1).^43^ As ^13^C line widths in frozen solution reflect the degree of residual conformational disorder of the side chains, the rather high overall resolution in this spectrum is explained by the well-defined rotamers for most Ile residues of GABARAP,^4^ which is typical for a globular protein, in contrast to an IDP.

However, the line widths for different Ile residues in GABARAP in frozen solution vary significantly (Table S1). Residues I32 and I64 exhibit extremely narrow lines (full width at half-maximum (fwhm) of 1.3 ± 0.1 and 1.4 ± 0.1 ppm, respectively) for all five side chain ^13^C atoms, which indicate particularly high conformational restriction for both residues. These two residues also exhibit low Cδ1 chemical shifts <10 ppm, which are typical for (g_m_,g_m_) rotameric conformations for both dihedral angles χ1 and χ2. Indeed, these conformations are also confirmed in the PDB structure 1KOT^42^ (Table 1). Both residues are located in well-defined regions of the protein: I32 in a β-sheet and I64 in an α-helix. Such a conformational restriction of the backbone in a defined secondary structure element may be a necessary but not sufficient prerequisite for the confinement of the side chain to the (g_m_,g_m_) rotamer. In contrast, for I21, located in an α-helix lining one of the two hydrophobic binding pockets, the single Cα–Cβ correlation visible at room temperature (Figure S1A) is split into two distinct Cα–Cβ cross-peaks at cryogenic temperatures (Figure 2C). One of these two signals has random-coil like^13^Cα shifts (63 ppm), while the other is shifted strongly toward α-helical chemical shifts (67 ppm). By integrating over the areas of I32, I64 and the split I21 peaks and relating them to the full signal volume (see Methods), we measured a fraction of the total signal volume of around 15% for each of them (Table S2). This result is in agreement with seven Ile residues (1/7 ≅ 0.14) contributing to the total integral. The I21 signal is split into two peaks, with an integral of about 7% each. This suggests that the^13^Cα chemical shift observed in solution at room temperature (64.9 ppm) represents an average between two rapidly exchanging states with roughly equal populations.

**Table 1:** Torsion Angles of the Isoleucine Side Chains in GABARAP, Measured in PyMOL Using the PDB File 1KOT(42)

**Ile**	**χ**_**1**_**angle (°)**	**state**	χ_**2**_**angle (°)**	**state**
21	–72.2	g_m_	151.7	trans
32	–44.4	g_m_	–53.4	g_m_
41	35.9	g_p_	177.1	trans
64	–64.6	g_m_	–55.3	g_m_
68	17.1	g_p_	162.9	trans
84	–56.6	g_m_	–152.9	trans
107	–59.1	g_m_	–160.6	trans

### Isoleucine in an Intrinsically Disordered Protein: α-Syn

**Figure 3:**
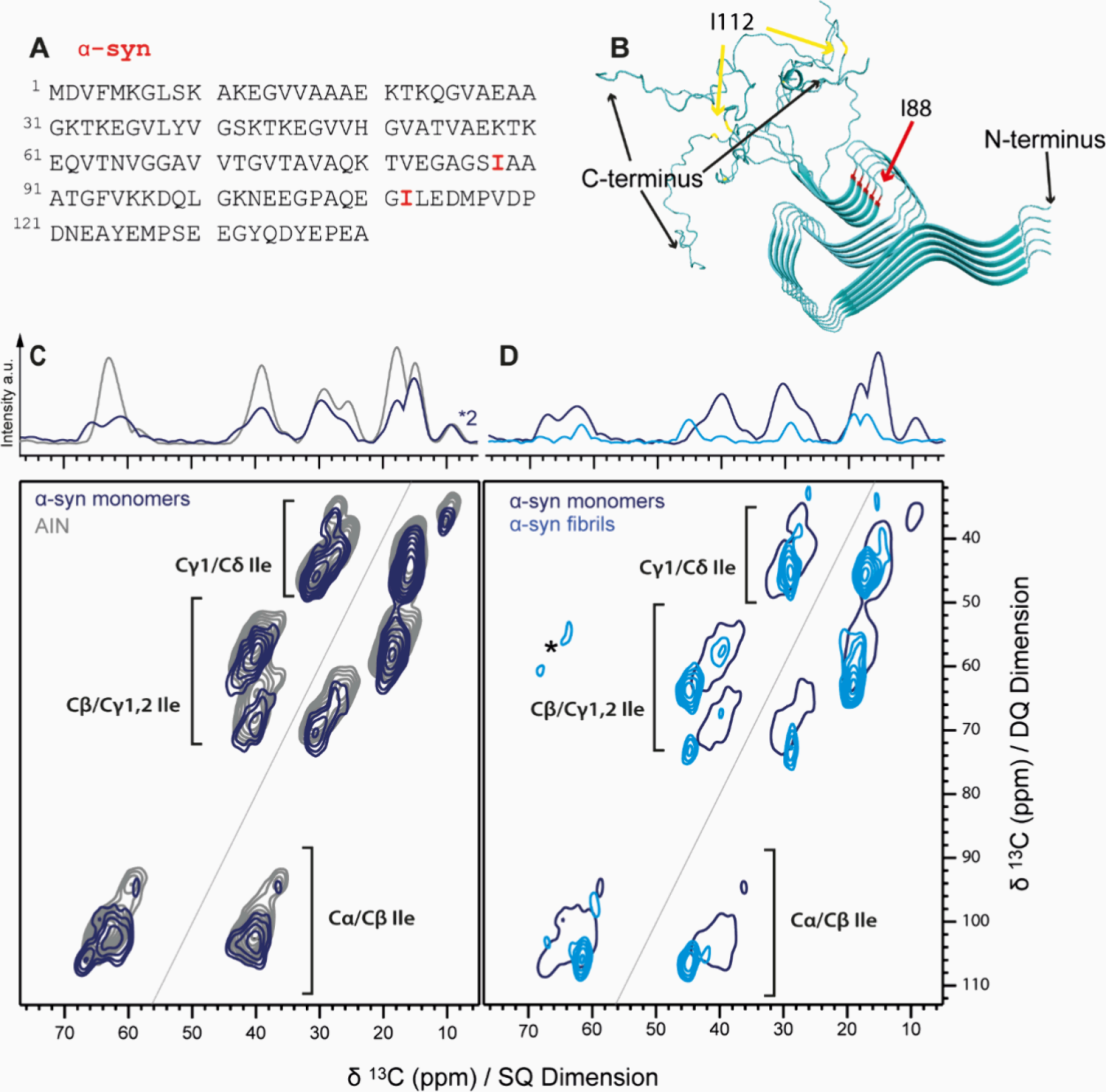
(A) Primary sequence of α-syn with both isoleucine I88 and I112 residues highlighted in red. (B) 3D structure of α-syn fibrils (PDB 2N0A) with the positions of I88 (cross-β core region) marked in red and I112 (flexible and therefore not confined region) in yellow.^44^ (C) 2D ^13^C–^13^C correlation DQ/SQ spectra of AIN (gray) and isoleucine-labeled monomeric α-syn (navy) recorded at cryogenic temperatures in frozen solution. (D) Isoleucine-labeled α-syn fibrils (blue) and the lowest contour level of α-syn monomers shown in (C) for comparison. Spinning sidebands of glycerol are marked with asterisks. 1D projections of the full spectra are shown on top of the spectra in (C) and (D).

To study the distribution of Ile side chain ^13^C chemical shifts in an IDP we selected α-syn. The primary sequence of α-syn has two Ile residues; one in the central amyloidogenic (formerly known as NAC) region (aa 61–95) at position 88, the other in the highly negatively charged C-terminus at position 112 (Figure 3A).

The DQ/SQ spectrum of monomeric α-syn shows relatively broad peaks (average fwhm of 3.6 ppm), indicating a very high degree of conformational freedom for the two Ile residues I88 and I112. The overlay of a DQ/SQ spectra of α-syn with that of the random coil mimetic AIN^20^ (Figure 3C) shows that the chemical shift range for Ile residues in α-syn roughly covers the full rotameric ensemble of an unfolded peptide mimetic. While chemical shift distributions for side chain carbon atoms agree well in both molecules, Cα chemical shifts of α-syn exhibit a larger dispersion than in AIN, with two distinct maxima around 66 and 62 ppm instead of one very broad maximum at 64 ppm, as observed for AIN. This finding reflects differences in the energy landscape for backbone conformations between a small peptide mimetic and a full protein. To quantify the amount of (g_m_,g_m_) conformation in Ile side chains and to obtain an estimate of the relative amount of α-helix like backbone conformations, respective peak areas were integrated and related to the full signal volume (see Methods). These integrated peak volumes from the α-syn DQ/SQ spectrum are in agreement with the peak volumes measured for AIN (Table 2 and Table S3) for (g_m_,g_m_) conformations, while the preference for α-helical conformations is slightly higher in monomeric α-syn.

**Table 2:** Populations of Different Conformations and Average Line Widths of Signals in Spectra of α-Syn and the Random Coil Peptide Mimetic AIN

	g_m_,g_m_population **(%)**	estimate of α-helical population**(%)**	**range of fwhm (ppm)**
AIN	14 ± 1	15 ± 1	2.2–3.2
α-syn (native)	15 ± 2	18 ± 2	2.7–4.9
α-syn (fibrils)	17 ± 6	9 ± 6	1.6–3.5

We thus could show that the side chains of both Ile residues in α-syn cover a broad conformational space, which is close to the conformational space in the random coil Ile mimetic AIN, while the backbone conformational space showed more pronounced conformational preferences toward either α-helical or extended conformations in α-syn. Overall, these results confirm the IDP character of α-syn.

For comparison, we recorded a DQ/SQ spectrum of Ile-labeled α-syn fibrils (Figure 3B) in frozen suspension (Figure 3D). The resonance pattern of the two Ile residues in the fibrillar form is dominated by peaks that are significantly narrower (see Table S4) than those of the monomeric IDP state and have chemical shifts typical for β-sheet secondary structure (Figure 3D). However, in addition, weak and broad signals covering a significant part of the typical random coil line shape pattern can also be observed. Even without knowing the exact fibril structure, we expect high β-sheet content for I88, as it is part of the fibril core in most fibril structures known so far.^44,45^ I112 on the other hand is expected to be disordered, as it is not inside the fibril core (Figure 3B) and thus gives rise to strongly inhomogeneously broadened signals. Although precise quantification of the signal patterns is challenging, the overall pattern is in line with a superposition of a well-defined I88 spin system with β-sheet secondary structure and a spin system from a disordered I112 residue.

### Different Stages in Protein Folding: PI3K SH3–Folded, Unfolded, and Fibrillar State

**Figure 4:**
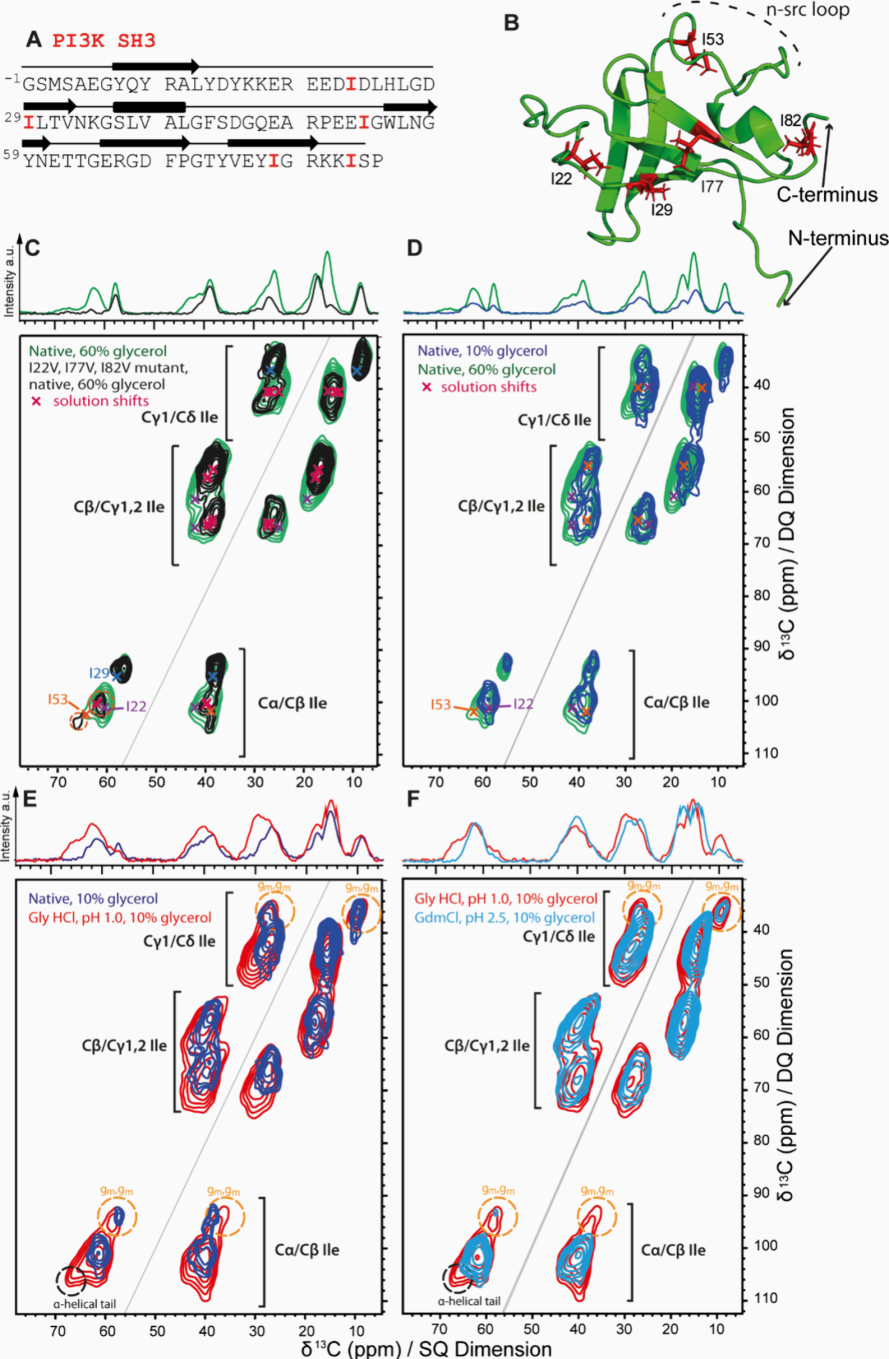
(A) Primary sequence of PI3K SH3 with the positions of isoleucine residues highlighted in red. The secondary structure elements α-helix and β-strand in the native structure are depicted as rectangles and arrows, respectively. (B) 3D structure of PI3K SH3 in its native form (1PNJ).^34^ The five Ile residues are labeled in red. (C) 2D ^13^C–^13^C correlation DQ/SQ spectra of isoleucine-labeled PI3K SH3 WT (green) and its I22V/I77V/I82V variant (black) in its native form recorded at cryogenic temperatures in frozen solution with 60% glycerol. The NMR chemical shifts of the natively folded protein in solution (Figure S2) are indicated by crosses. The peak doubling experienced by I53 is highlighted with orange circles. (D) Comparison between 2D ^13^C–^13^C correlation DQ/SQ spectra of isoleucine-labeled PI3K SH3 in its native form recorded at cryogenic temperatures in frozen solution with 60% glycerol (green) and 10% glycerol (navy blue). (E) Comparison of isoleucine-labeled PI3K SH3 in its native form in 10% glycerol (navy blue) with its unfolded state at pH 1.0 (red). (F) Comparison between DQ/SQ spectra of Ile-labeled PI3K SH3 at pH 1.0 (red) and at pH 2.5 in the presence of 6 M GdmCl (light blue). 1D projections of the 2D spectra are shown on top.

To investigate different stages of protein folding, unfolding and misfolding, we chose the bovine PI3K SH3 domain (Figure 4A), which differs from its human homologue p85α PI3K SH3 by only one residue in position 49. This SH3 domain is a promising model system, as it can adopt many different (meta)stable states depending on pH, buffer conditions and temperature. At neutral pH, the protein adopts the stable five-stranded antiparallel β-sandwich fold typical of SH3 domains^34−37^ (Figure 4B).

**Figure 5:**
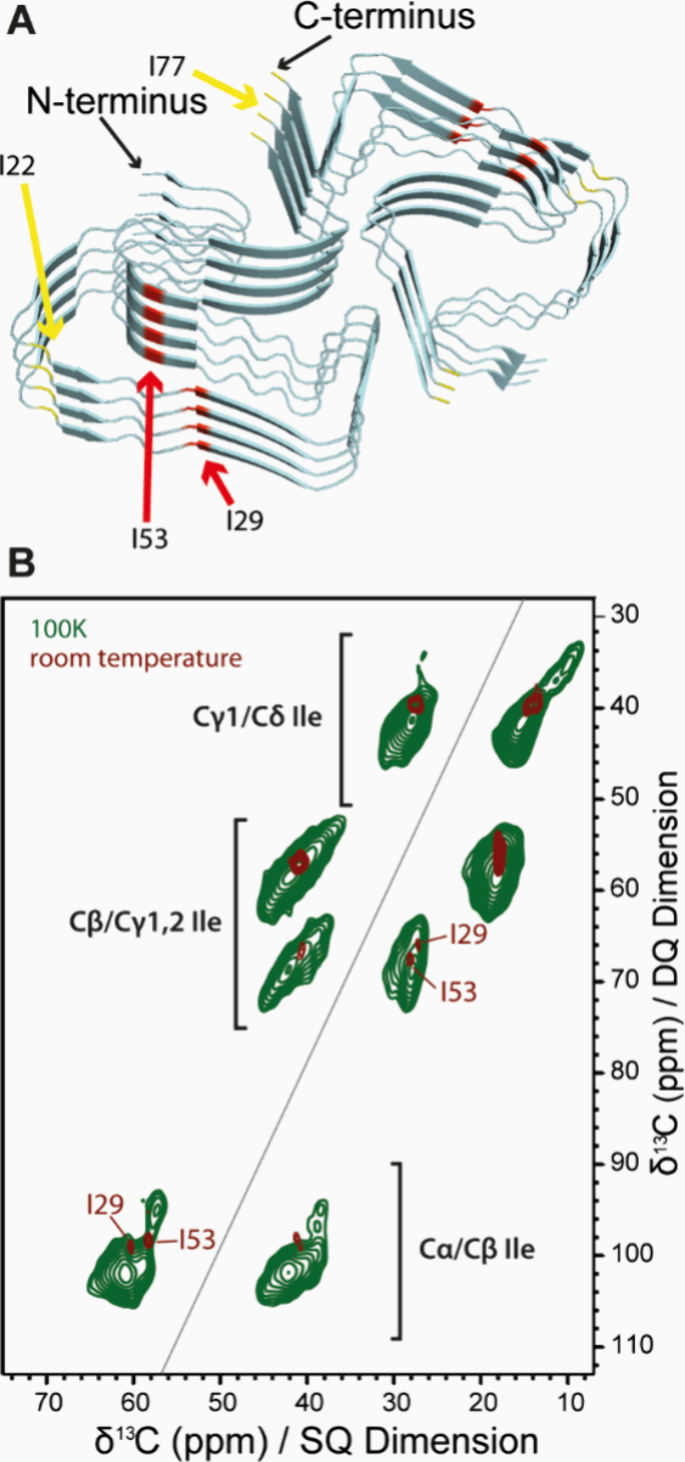
(A) 3D structure of PI3K SH3 fibrils (6R4R)^41^ spanning the sequence region from M1 to I77, with the isoleucine residues highlighted in red (fixed region) and yellow (flexible region). The disordered C-terminus including I82 is not part of the structural model deposited with the PDB. (B) 2D^13^C–^13^C DQ/SQ spectrum of isoleucine-labeled PI3K SH3 in its fibrillar form in frozen solution (green) overlaid with that of fibrillar PI3K SH3 recorded at ambient temperature (brown).

Compared to most SH3 domains from the src tyrosine kinase family, however, the n-src loop connecting strands β2 and β3 is approximately 15 residues longer in the PI3K SH3 and somewhat mobile, as reflected in reduced^46^^15^N NOE values.^34^ At acidic pH the PI3K SH3 domain (partially) unfolds, and in this unfolded state, it is also prone to fibril formation.^33^ The structure of the amyloid fibrils formed at pH 2.5 has recently been determined by cryoEM.^41^ In this fibril type, almost the entire protein is part of the well-defined parallel in-register β-sheet core (Figure 5A). We used the five isoleucine residues in the PI3K SH3 domain (Figure 4A) as reporters for backbone and side chain flexibility in the native, unfolded and fibrillar state in frozen solution.

### Native State and Unfolded States

The PI3K SH3 domain is well-ordered at neutral pH. In the native form, two isoleucines are located in β-strands β2 (I29) and β5 (I77) (Figure 4A). I22 is located in the relatively rigid hairpin-like RT-src loop connecting strands β1 and β2 (Figure 4B). I53 is located in a helical loop at the end of the somewhat mobile n-src loop, while I82 is part of the C-terminal tail, which is disordered.^34^ The DNP spectrum recorded in frozen buffer solution containing 60% glycerol is shown in Figure 4C and agrees well with the chemical shifts obtained by solution NMR spectroscopy (Figure S2), which in turn are similar to the chemical shifts published previously for bovine PI3K SH3.^47^ However, in frozen solution, the chemical shift dispersion of the five PI3K SH3 isoleucine residues is insufficient to resolve individual resonances due to inhomogeneous line broadening which is larger than the shift dispersion for most of the residues. Consequently, most signals overlap in the DNP spectrum, forming a composite set of cross-peaks. This observation aligns with the domain’s structure, as PI3K SH3 contains isoleucine residues (e.g., I83) within less ordered regions. Notably, I29, located within a β-sheet (strand β2), exhibits a characteristic low Cδ chemical shift and distinctive Cα and Cβ shifts in solution NMR, indicative of the (g_m_,g_m_) side chain conformation, consistent with that observed in most PI3K SH3 PDB structures (Table 3). These shifts are clearly visible in both solution (Figure S2) and frozen solution spectra (Figure 4C). To confirm this assignment, we expressed a PI3K SH3 sequence variant where I29 is replaced by the also β-branched amino acid valine (Figure S3A), a point mutation, which does not perturb the protein structure, as confirmed by solution NMR (Figure S3C)**.** The absence of the well-resolved (g_m_,g_m_) signals in the corresponding spectrum in frozen solution of this variant unambiguously identifies these signals as originating from I29 (Figure S4A)**.**

**Table 3:** Rotameric States of the Isoleucine Side Chains in the Human PI3K SH3 Determined in PyMOL Using the PDB Files 2PNI,^34^ 3I5S,^37^ 1PKT,^35^ 1PHT,^36^ and 6R4R^41^^a^

	**2PNI (NMR, 26 models)**		**3I5S (X-ray, 4 subunits)**		**1PKT (NMR, 30 models)**		**1PHT (X-ray)**		**6R4R (fibril, CryoEM)**	
residue	χ1	χ2	χ1	χ2	**χ**_**1**_	**χ**_**2**_	χ1	χ2	χ1	χ2
I22	g_p_	t	g_p_	t	g_p_	n.d.	g_p_	t	g_m_	t
I29	g_m_	n.d.	g_m_	g_m_	g_m_	n.d.	g_m_	g_m_	g_m_	t
I53	g_m_	t	g_m_	t	g_m_	t	g_m_	t	g_p_	t
I77	g_p_	t	g_p_	t	n.d.	n.d.	g_p_	t	g_m_	t
I82	n.d.	n.d.	n.d.	n.d.	n.d.	n.d.	g_m_*	g_m_*	n.d.	n.d.

a Dihedral angles whose circular variance over the ensemble of models/subunits exceeds 0.1 were considered ill-defined (n.d.). I82 is not included in models 3I5S, 1PKT, and 6R4R; in the model 1PHT, it is constrained by an artificial crystal contact (indicated by *).

To unambiguously identify the signals of I53, which is located in a helical turn in the mobile n-src loop (Figure 4B), we expressed a second sequence variant, where I22, I77 and I82 were replaced with valine (Figure S3B). Also in this variant, the overall structure of the PI3K SH3 domain was conserved (Figure S3D). The DNP spectrum of this mutant displays two distinct Cα/Cβ cross peak signals for I53, in addition to the (g_m_,g_m_) peak of I29 (Figure 4C and Figure S5A). Thus, I53, like I21 in GABARAP, exhibits a doubling of the Cα resonance. This observation is consistent with I53’s location within a helical turn in the mobile n-src loop (Figure 4B).

Since some of the five Ile residues are located in regions with enhanced flexibility, the range of backbone as well as of side chain conformations are expected to be sensitive reporters on the effect of changes in solution conditions.

To study the effect of pH and solvent conditions on the conformational ensemble adopted by PI3K SH3 in more detail, we also performed a series of DNP experiments in different buffers containing only 10% glycerol as a cryoprotectant. Decreasing the glycerol concentration in the solvent from 60% to 10% significantly narrows the chemical shift range of inhomogeneously broadened signals in phosphate buffer at pH 6.8 (Figure 4D). Notably, the minor peak characteristic of α-helical propensity, previously observed for the Cα/CαCβ cross-peak (64–66 ppm of SQ and 100–110 ppm DQ) at 60% glycerol, is greatly diminished at 10% glycerol. This reduction in α-helical signal is also evident in the I22V/I77V/I82V variant for Ile53 (Figure S5A), for which the estimate of α-helical propensity is reduced (Table S5).

Moreover, in the spectra of the WT-PI3K SH3 domain, the range of Cβ and Cγ1 shifts is reduced at 10% glycerol concentration. In particular, the Cβ and Cγ1 chemical shifts of I22 in native solution (indicated as crosses in Figure 4D) are no longer covered by the inhomogeneous peak shapes at 10% glycerol, indicating that an amount of 60% glycerol indeed helps to stabilize native conformations in proteins at cryogenic temperature. Therefore, 10% glycerol were used to study unfolded states of PI3K SH3.

In frozen solution in a pH 2.5 buffer with 10% glycerol, the overall appearance of the spectrum does not differ substantially from that obtained at pH 6.8 (Figure S6), suggesting that the native fold is not affected by this pH value at temperatures close to the freezing point. By contrast, in liquid solution at pH 2.5 the PI3K SH3 domain is in a temperature-dependent equilibrium between the native state and a denatured state, the latter becoming the dominant form at temperatures above 40 °C (99% denatured form).^33^ Lowering the pH further to a value of 1.0 leads to a substantial broadening of all lines in the low-temperature spectrum (Figure 4E; average fwhm of 4.09 ppm), suggesting that the backbone conformational space is enhanced due to the lack of secondary structure restraints. At pH 1.0, the Cα/Cβ cross peak covers the typical chemical shift range of IDPs (see Figure 3C), including even α-helical chemical shifts. Furthermore, the reduced intensity of peaks characteristic of the (g_m_,g_m_) conformation, compared to folded PI3K SH3, indicates a loss of structural constraint for I29. This suggests that, at this pH, single conformation restrictions are abolished, allowing each residue to explore its entire conformational landscape. This conclusion is supported by analyses of the I29V (Figure S4B) and I22V/I77V/I82V (Figure S5B) variants (Table S5).

The PI3K SH3 can alternatively be unfolded by addition of 6 M guanidine hydrochloride (GdmCl) at pH 2.5 (Figure 4F). While the overall line-width for most resonances is as broad as for unfolded PI3K SH3 at pH 1.0, some significant differences are observed: (i) The Cα/Cβ cross peaks are devoid of any α-helical population, and (ii) the (g_m_,g_m_) population is lower than for PI3K SH3 at pH 1.0. This finding indicates that GdmCl shifts the equilibrium of backbone conformations toward extended conformations.

We quantified the relative amounts of Ile in (g_m_,g_m_) conformation and an estimate the population with α-helical backbone conformation by integrating the indicative peak areas (Figure S7 and Tables S3 and S6) for PI3K SH3 in all states. Once again, we employed the random coil mimic AIN for comparison. In AIN, the (g_m_,g_m_) population as determined from the Cα/Cβ and Cγ1/Cδ1 cross peaks is between 15% and 11%, respectively, the estimate of α-helical population determined from the Cα/Cβ cross peak is roughly 15% (Table 2). Interestingly, the populations of the (g_m_,g_m_) state as well as the α-helix population of the combined signals of all five Ile residues in unfolded PI3K SH3 at pH 1.0 are very similar to those of AIN, suggesting that backbone torsion angles as well as side chain rotamers of all five Ile residues of denatured PI3K SH3 at pH 1.0 cover the same conformational space as in the random coil peptide mimic AIN.

For natively folded PI3K SH3 at pH 6.8, the α-helical population is close to 0%, reflecting the fact that no Ile residue is located in an α-helix. The population of the (g_m_,g_m_) conformation is between 23% and 25% and thus higher than in random coil (∼15%). I29 adopts the (g_m_,g_m_) conformation in solution (see above) and is therefore expected to adopt the (g_m_,g_m_) conformation with a population close to 100% in frozen solution as well. The contribution of I29 to the (g_m_,g_m_) area of the combined signal of all five Ile residues is therefore estimated to be 20%. The observation that the (g_m_,g_m_) signal contribution is higher than 20% is thus an indication that other Ile residues may as well in part adopt the (g_m_,g_m_) conformation. In particular, I82 in the disordered C-terminus should have sufficient conformational freedom to populate the (g_m_,g_m_) rotamer to some degree.

Upon lowering the pH to 2.5, the (g_m_,g_m_) population decreases to ∼20%. Since restriction of I82 and I22 to (g_p_,t) or (t,t) rotamers is implausible, the lower overall population of (g_m_,g_m_) likely reflects more extensive conformational freedom of the side chain of I29 to also populate other rotamers at pH 2.5 as the native hydrophobic core packing is destabilized. This is also supported by the rise of the α-helical population to 7% (Table 4), suggesting increased flexibility of the protein at pH 2.5. This trend continues and culminates in full conformational flexibility in the denatured protein at pH 1.0, where α-helical as well as (g_m_,g_m_) populations resemble those in an IDP (Tables 2 and 4).

**Table 4:** Populations of Different Conformations and Average Line Widths of Signals in Spectra of Bovine PI3K SH3 in Different Solvents

**PI3K SH3**	g_m_,g_m_population **(%)**	index of α-helical population **(%)**	**range of fwhm (ppm)**
native, 60% glycerol (pH 6.8)	25 ± 1	4 ± 1	2.0–3.6
native, 10% glycerol (pH 6.8)	23 ± 1	1 ± 1	2.0–4.6
fibrils, 60% glycerol (pH 2.5)	11 ± 4	6 ± 4	1.6–3.5
intermediate, 10% glycerol (pH 2.5)	20 ± 2	7 ± 2	3.3–5.8
unfolded, 10%glycerol (pH 1.0)	16 ± 2	16 ± 2	2.8–6.2
unfolded, GdmCl, 10% glycerol (pH 2.5)	8 ± 1	0 ± 1	2.9–5.2

For comparison, we also recorded and analyzed PDSD spectra of PI3K SH3 at different conditions (see Figure S8 and Table S7). Evaluation of respective Cα/Cβ cross peaks gave similar results within error margins (Cγ1/Cδ1 cross peaks could not be evaluated as they overlap with Cγ1/Cγ2 relay cross peaks). For the estimate of α-helical populations, a similar trend is observed.

### PI3K SH3 Fibrils

Ile-labeled PI3K SH3 amyloid fibrils formed at pH 2.5 (Figure 5A) were studied both at room temperature and in frozen suspension. The comparison between DQ/SQ spectra of Ile-labeled PI3K SH3 fibrils recorded in the different conditions is shown in Figure 5B. At room temperature, only I29 and I53 give rise to cross peaks, while I77 and I82 at the disordered C-terminus of the protein and I22 in a stretch with enhanced mobility (unpublished results) are apparently undetectable. Secondary chemical shifts of I29 and I53 in PI3K SH3 fibrils are typical for β-sheet backbone conformation and their Cδ1 shifts are typical for a χ2 angle in trans orientation (Figure 5B).

At cryogenic temperatures, the mobility of these three residues is frozen out, and the five Ile residues give rise to overlapping heterogeneously broadened signals (Figure 5B). The intensities of the Cγ1/Cδ1 cross peaks typical for the (g_m_,g_m_) rotamer are clearly visible, with intensities of ∼11% of the total signal, indicating that the (g_m_,g_m_) state is still accessible for at least some of the Ile residues in the fibrils (in particular I77 and I82 and possibly also I22), but the percentage is significantly reduced compared to natively folded and unfolded monomeric PI3K SH3 states. Moreover, the α-helical conformation represents about 6% of the population. With an α-helical propensity of 15% for an Ile residue in an IDP (Table 2), this result is in agreement with two out of five Ile residues (I77 and I82) being completely disordered.

## Conclusions

In this study, we introduce a novel NMR-based approach to assess the conformational dynamics of proteins by analyzing backbone conformational and rotameric ensembles of isoleucine side chains. To this end, we selected several proteins that cover a variety of different stages in protein folding, from disordered and denatured states to well-folded native states and to misfolded amyloid fibrillar states.

In well-folded proteins, rigid structural constraints limit backbone and side chain motion, resulting in well-resolved NMR signals for individual isoleucine residues at cryogenic temperatures.^48^

For the protein GABARAP in frozen solution, we could discriminate individual signals for most of the seven Ile residues, whereas for well-folded bovine PI3K SH3 the signal overlap in frozen solution was larger, as three out of five Ile residues in this domain are located at the flexible C-terminus or in coil regions lacking well-defined secondary structure. However, with the help of two variants with one and three Ile to Val point mutations, we could identify individual signals also in this protein. In both proteins, individual line shapes of different Ile residues differ strongly between the residues, and the narrowest lines could be observed for those residues, which are confined in (g_m_,g_m_) conformation, i.e. I32 and I64 in GABARAP and I29 in PI3K SH3. In both proteins, these residues are located in well-defined secondary structure elements, which is linked to steric confinement of the side chains. In both proteins, for one Ile residue each located in an α-helix a signal doubling for Cα was observed (I21 in GABARAP, I53 in PI3K SH3) at low temperature. This indicates that the chemical shifts observed for these positions at room temperature are the result of residual conformational averaging, which takes place even in well-defined globular proteins.

In unfolded and intrinsically disordered proteins backbone and side chain conformations can rapidly interchange at ambient temperature, thus leading to an averaging of secondary chemical shifts or the characteristic low-frequency shift of Cδ1 of Ile in the (g_m_,g_m_) conformation. Therefore, the chemical shift dispersion between different Ile residues in unfolded proteins is usually low. In frozen solution, the large distribution of chemical shifts leads to substantial inhomogeneous line broadening which dominates the line shape. We could demonstrate for the monomeric IDP state of α-syn that the two Ile residues I88 and I112 in the unfolded monomer adopt the same conformational space as in random coil peptide mimetics. The typical IDP-like spectrum is obtained for PI3K SH3 at pH 1.0, when the protein is completely unfolded. Under these acidic conditions, the conformational freedom for Ile side chains is similar to that in an IDP and in the random coil peptide mimetics. A different unfolded state of PI3K SH3 is obtained when the protein is denatured by a strong unfolding agent such as GdmCl in high concentration. This denaturant appears to stabilize the backbone in an extended conformation where, despite of the protein’s high backbone flexibility, helical conformations are prevented.

In the fibrillar state, in α-syn as well as in PI3K SH3 most Ile residues are conformationally restricted as part of the β-sheet fibril core, which is reflected by chemical shifts typical for β-sheet secondary structure dominating the resonance pattern in the spectra, as well as a reduced population of the (g_m_,g_m_) conformation. However, in both fibril types, not all Ile residues are part of the β-sheet core. In α-syn the C-terminal I112 residue, and in PI3K SH3 fibrils I77 and I82 remain disordered as part of the fuzzy coat outside the fibril core, and give rise to inhomogeneously broadened signals covering the typical random coil line shape.

For each protein, by integrating the peaks’ volumes, we were able to quantify the prevalence of selected conformations. This approach matched with the respective PDB files and provided further information. In particular by successfully elucidating different unfolded states of PI3K SH3, we demonstrated that unfolded proteins can cover different conformational spaces in different solvents.

Future outlooks of this study include the extension of the investigation to amino acids other than isoleucine. Moreover, the presumed impact of glycerol on the conformation of the protein as well as the possible structural reorganization during the sample freezing process should be further inspected.

In conclusion, our study highlights the strength of NMR in tackling one of the major challenges in the structural investigation of (partially) disordered biomolecules. By cooling the sample to cryogenic temperatures, we stop the physiological conformational exchange and take a snapshot of the investigated molecules. Thus, all conformations sampled by each nucleus are preserved in line with their respective probability rather than average values.

## Material and Methods

### Expression and Purification of Ala and Ile-Labeled GABARAP

Expression and purification of GABARAP protein were carried out essentially as described.^4^ However, in case of selectively Ile-labeled protein, the expression protocol had minor changes. GABARAP protein was expressed recombinantly in *E. coli* BL21(DE3)-T1R in 1 L M9 minimal medium. The medium contained [^13^C,^15^N]-Ile and [^13^C,^15^N]-Ala as the only labeled amino acids (Cortecnet) at a concentration of 150 mg/L. The other 18 proteinogenic amino acids (Sigma-Aldrich) were added in natural isotope abundance at concentrations of 150 mg/L. The cultures were induced with 1 mM Isopropyl β-D-1-thiogalactopyranoside (IPTG) after reaching an OD_600_ of 0.8 and grown for further 15 h at 20 °C before harvesting.

### Expression and Purification of Ile-Labeled α-Syn

Selectively isoleucine-labeled N-terminal acetylated α-syn_1–140_ was expressed in *Escherichia coli* BL21(DE3) carrying codon-optimized α-syn in pT7 vector and the pNatB vector with the N-terminal acetylation enzyme from *Schizosaccharomyces pombe*.^49,50^ M9 medium was supplemented with all 19 proteinogenic nonlabeled amino acids and 150 mg/L [^13^C, ^15^N]-labeled isoleucine, similar to a protocol described previously.^51^ Protein purification was performed as described previously.^52^ The culture was grown at 37 °C with 120 rpm shaking, at OD_600_ of 1.2 α-syn expression was induced with 1 mM IPTG, and cells were harvested after 6 h at 37 °C. Cell pellets were resuspended in 25 mL 20 mM Tris pH 8.0 and boiled at 95–100 °C for 2 × 15 min. After centrifugation at 20.000*g* for 30 min at 4 °C recombinant α-syn was precipitated from the supernatant using a final concentration of 0.45 g/mL ammonium sulfate and centrifuged at 20.000*g* for 30 min. The pellet was dissolved in 50 mL 20 mM Tris-HCl pH 8.0 and loaded on a HiPrep Q Fast flow 16/10 anion exchange column 16/10 (Cytiva, CV = 20 mL). Elution was performed by applying a linear NaCl gradient of 20-fold CV from 0 mM to 800 mM NaCl in 20 mM Tris-HCl pH 8.0. α-syn was eluted at a conductivity of 28–32 mS/cm and fractions containing α-syn were pooled and precipitated using ammonium sulfate, as described previously. The protein pellet was resuspended in 5 mL 50 mM Tris-HCl pH 7.4 and loaded on a HiLoad Superdex 60/75 pg SEC column (Cytiva, CV = ∼120 mL). α-syn eluted at ∼60 mL with a final yield of ∼8 mg/L culture and >97% purity.

### Expression and Purification of Ile-Labeled PI3K SH3

The Ile-labeled WT-PI3K SH3 domain from *Bos taurus* was expressed in *E. coli* BL21 (DE3) ROSETTA with an additional His_6_-tag for purification followed by a thrombin protease cleavage site.^41^ Cells were grown in M9 medium supplemented with all 19 proteinogenic nonlabeled amino acids and 150 mg/L [^13^C, ^15^N]-labeled isoleucine for 24 h. Gene expression was induced by 1 mM IPTG at an OD_600_ of 0.6. Cells were harvested by centrifugation and resuspended in 50 mM HEPES/NaOH buffer, pH 7.6, containing 100 mM NaCl, 0.3 mM phenylmethylsulfonyl fluoride (PMSF), 20 mg/L DNase I and 70 mg/L lysozyme. After sonication on a Bandelin sonopuls sonicator using a VS 70T sonotrode (60% amplitude, 3× 5 min, 3 s ‘on’, 5 s ‘off’) on ice and ultracentrifugation in a Beckman Optima XPN-80 ultracentrifuge equipped with a 70Ti rotor at 4 °C and 42.000 rpm for 1 h. The supernatant was loaded on 5 mL Protino Ni-NTA column (Macherey-Nagel, Düren, Germany) equilibrated with 50 mM HEPES/NaOH, 100 mM NaCl and 20 mM imidazole, pH 7.6. The protein was eluted in a linear imidazol gradient (from 20 mM to 500 mM imidazole in 50 mM HEPES/NaOH, 100 mM NaCl, pH 7.6 within 20 CV). The fractions containing the eluted protein were pooled, concentrated and the His_6_-tag was cleaved off with thrombin using 5 U thrombin (SERVA Electrophoresis GmbH, Heidelberg, Germany) per mg protein for 2 days at 4 °C under mild shaking conditions. The protein solution was loaded on a SEC HiLoad 16/60 Superdex 75 column (GE Healthcare Europe GmbH, Freiburg, Germany) equilibrated with 5 mM ammonium acetate, pH 7.7. Fractions containing PI3K SH3 were pooled and lyophilized before further usage. As described previously [29], the sequence of the bovine PI3K SH3 construct contains a glycine-serine overhang at the N-terminus from the thrombin cleavage site and has the amino-acid sequence GS MSAEGYQYRA LYDYKKEREE DIDLHLGDIL TVNKGSLVAL GFSDGQEAKP EEIGWLNGYN ETTGERGDFP GTYVEYIGRK KISP.

The bovine PI3K SH3 variants were expressed and purified essentially as described in ref (39). Briefly, bovine PI3K SH3 domain was expressed in *E. coli* BL21 (DE3) as a GST-fusion protein using the pGEX-4T vector. Cells were grown in isotope-labeled M9 medium containing all 19 proteinogenic nonlabeled amino acids and 150 mg/L [^13^C,^15^N]-labeled isoleucine, lysed, and the fusion protein was purified using GST-affinity chromatography. The GST tag was cleaved with thrombin and the cleaved PI3K SH3 domain was further purified via an additional GST-affinity step to remove residual GST and size exclusion chromatography. The final [U–^13^C,^15^N]-labeled protein was lyophilized before further usage.

The amino acid sequence of the I29V variant is GS MSAEGYQYRA LYDYKKEREE DIDLHLGD**V**L TVNKGSLVAL GFSDGQEAKP EEIGWLNGYN ETTGERGDFP GTYVEYIGRK KISP, and the amino acid sequence of the I22V/I77V/I82V variant GS MSAEGYQYRA LYDYKKEREE D**V**DLHLGDIL TVNKGSLVAL GFSDGQEAKP EEIGWLNGYN ETTGERGDFP GTYVEY**V**GRK K**V**SP.

### Fibril Formation of Ile-Labeled α-Syn

α-Syn fibril formation was performed by incubating 100 μM purified monomeric α-syn in PBS (137 mM NaCl, 2.7 mM KCl, 10 mM Na_2_HPO_4_, 1.8 mM NaH_2_PO_4_), 0.05% (w/v) NaN_3_ pH 7.4 at 37 °C and 900 rpm continuous shaking in a sealed 2 mL low-bind surface reaction tube (Eppendorf, GE). For induction of aggregation one borosilicate bead was added (*d* = 3 mm, Hilgenberg, GE). Mature α-syn fibrils were harvested after five days incubation at 100.000 × *g* for 30 min at 4 °C and the total α-syn fibril mass was determined by subtracting the concentration of the soluble protein fraction found in the supernatant. The α-syn fibril pellet was washed several times with a stock solution of H_2_O/D_2_O buffer mixture in a 1:3 ratio after ultracentrifugation using a TLA-55 Fixed-Angle Rotor (100.000*g*).

### Fibril Formation of Ile-Labeled PI3K SH3

100 nmol lyophilized Ile-labeled PI3K SH3 were dissolved in 1 mL of 10 mM glycine/HCl buffer (pH 2.5) prepared from 20 μL 500 mM glycine/HCl buffer (pH 2.5) and 980 μL D_2_O. Unlabeled fibril seeds were added to a final concentration of 5 μM (monomer concentration and the solution was incubated at 50 °C overnight under quiescent conditions in an Eppendorf tube, as described before.^41^

### DNP Sample Preparation

AIN, GIG, Ala-Ile-labeled GABARAP and Ile-labeled α-syn samples for DNP-enhanced NMR prepared in d8-glycerol/D_2_O/H_2_O solutions (60:30:10 volume ratio) with 2.5 mM AMUPol or 5 mM M-TinyPol. As a first step, a buffer containing 150 mM NaCl, 100 mM NaPi, and 30 mM NaN_3_ in a H_2_O/D_2_O mixture at a ratio of 1:3 ratio was prepared, and each protein sample was buffer-exchanged to this buffer using Amicon centrifugal filter devices with a 3 kDa cutoff. The sample was then concentrated to 10–12 μL. d8-glycerol was added to a final concentration of 60% (v/v). Finally, 2.5 mM AMUPol or 5 mM M-TinyPol was added from a 100 mM stock solution.

For the DNP sample preparation of α-syn fibrils, after the buffer exchange and concentration of the sample via ultracentrifugation, d8-glycerol was added to a final concentration of 60% (v/v). Finally, 2.5 mM AMUPol or 5 mM M-TinyPol was added from a 100 mM stock solution.

For Ile-labeled PI3K SH3 in its native state, lyophilized protein (100 nmol) was dissolved in either (i) 75 μL 60:30:10, d8-glycerol/D_2_O/H_2_O v/v/v, containing 25 mM NaPi, pH 6.8, or (ii) 67 μL 10:80:10, d8-glycerol/D_2_O/H_2_O containing 25 mM NaPi, pH 6.8. For unfolded conditions, the protein was dissolved in (iii) 10:80:10, d8-glycerol/D_2_O/H_2_O v/v/v, containing 50 mM glycine/HCl, pH 2.5 or 1.0, or (iv) 10:80:10, d8-glycerol/D_2_O/H_2_O v/v/v containing 6 M GdmCl, pH 2.5. M-TinyPol was added to a final concentration of 5 mM, yielding to a final protein concentration of ∼1.4 mM.

For the fibrillar sample of PI3K SH3 the fibril solution was centrifuged at 186.000 × *g* in an OptimaTM MAX-XP ultracentrifuge equipped with a TLA-55 rotor for 1 h at RT. The supernatant was removed and the fibril pellet (∼15–20 μL for 100 nmol monomer equivalent) was resuspended 60:30:10, d8-glycerol/D_2_O/H_2_O v/v/v, containing 10 mM glycine/HCl and 5 mM M-TinyPol.

All samples were filled into 3.2 mm sapphire rotors, and measurements were performed at 100 K.

### Solution NMR Spectroscopy

A sample of 0.50 mM [^13^C,^15^N]-Ala/Ile *H. sapiens* GABARAP with 100 mM NaCl, 100 mM KCl, 0.1 mM EDTA, 25 mM sodium phosphate buffer in 10% (v/v) D_2_O (pH 6.9) was used for solution NMR spectroscopy. Solution NMR measurements of the *B. taurus* PI3K SH3 at neutral pH were performed on samples containing 0.25 mM [U–^13^C,^15^N] PI3K SH3 wt, 0.25 mM [^13^C,^15^N]-Ile PI3K SH3 wt, or 0.25 mM [^13^C,^15^N]-Ile PI3K SH3 I29V with 25 mM sodium phosphate buffer in 8% (v/v) D_2_O (pH 6.8) at a temperature of 25.0 °C. Acidic samples contained 0.28 mM [U–^13^C,^15^N] or 0.40 mM [^13^C,^15^N]-Ile *B. taurus* PI3K SH3 wt with 10 mM glyine/HCl buffer in 8% (v/v) D_2_O (pH 2.5). 2D ^1^H–^15^N HSQC spectra^53^ were recorded to verify the integrity of the samples after lyophilization and dissolution in the respective buffer. Two 2D ^13^C–^13^C TOCSY spectra covering either the aliphatic (bandwidth 70 ppm) or full (bandwidth 180 ppm) spectral region with a 13.6 ms (aliphatic) or 20.4 ms (full bandwidth) 13.9 kHz FLOPSY-16 isotropic mixing scheme^54^ were recorded on the [^13^C,^15^N]-Ala/Ile GABARAP sample on a Bruker AVANCE III HD 600 MHz NMR spectrometer equipped with a cryogenically cooled inverse quadruple resonance probe. For each PI3K SH3 sample, aliphatic and full bandwidth (not recorded on [^13^C,^15^N]-Ile PI3K SH3 at pH 2.5) 2D ^13^C–^13^C TOCSY spectra with a 15.1 ms (aliphatic) or 21.1 ms (full bandwidth) 15.6 kHz FLOPSY-16 isotropic mixing scheme^54^ were recorded on a Bruker AVANCE III HD 800 MHz NMR spectrometer equipped with a cryogenically cooled ^13^C/^15^N observe triple resonance probe. All probes had *z* axis pulsed field gradient capabilities. The sample temperature was calibrated using methanol-d_4._^46^ Quadrature detection in the indirect ^13^C dimension was achieved by States-TPPI.^55^ All solution NMR spectra were processed with NMRPipe^56^ software and analyzed with NMRViewJ^57^ and CCPN.^58,59^^1^H chemical shifts were referenced with respect to external DSS in D_2_O, ^13^C and ^15^N chemical shifts were referenced indirectly.^60^

### DNP Experiments

All experiments were conducted on an 18.8 T (800 MHz ^1^H Larmor frequency) spectrometer (Bruker Avance) connected to a 525 GHz gyrotron as a source of continuous microwaves for cross-effect DNP hyperpolarization. Enhancement factors obtained were between 19 and 64 (see Figure S3 and Tables S8 and S9). All samples were filled into 3.2 mm sapphire rotors. All experiments were performed at a temperature of 100 K. Spectra were recorded using a recycle delay of 5 s. The 2D ^13^C–^13^C double quantum/single quantum (DQ/SQ) SPC5 spectra were recorded using a magic angle spinning frequency of 8.2 kHz and SPC5 recoupling.^61^ SPC 5 recoupling time and reconversion times were set to 488 μs in all experiments, corresponding to four rotor periods. Proton Driven Spin Diffusion (PDSD) spectra were recorded at MAS frequencies of 11 kHz (GABARAP) or 12 kHz (PI3K SH3) with mixing times between 10 and 50 ms. Contact times for cross-polarization were between 100 and 900 μs. High power proton decoupling with an rf power of ∼83 kHz was applied during evolution and detection as well as DQ excitation and recoupling. During SPC5 recoupling, CW proton decoupling was employed, during evolution and detection SPINAL-64 decoupling.^62^

Experimental parameters for all samples, including number of scans, t_1_ increments, maximum evolution time and total experimental time are provided in Tables S8 and S9. Spectra were referenced externally using adamantane by calibrating its CH peak to 31.4 ppm, corresponding to the DSS reference scale. All NMR spectra were processed in TopSpin 3.6-4.3 and analyzed using CCPN. Relevant processing parameters of the spectra are listed in Table S10. DQSQ spectra were plotted with contour lines at 1.31 incremental spacing, PSDS spectra with an increment of 1.4.

### Solid-State MAS NMR Spectroscopy at Ambient Temperature

The DQSQ spectrum of Ile-labeled PI3K SH3 fibrils was recorded on a Bruker 600 MHz spectrometer equipped with an AVANCE NEO console and a 3.2 mm triple resonance probe head. VT gas was adjusted to −10 °C, resulting in an effective sample temperature of ∼0 °C.

The 2D ^13^C–^13^C double quantum/single quantum (DQ/SQ) SPC5 spectrum was recorded using a magic angle spinning frequency of 8.0 kHz and SPC5 recoupling.^61^

High power proton decoupling with an rf power of ∼83 kHz was applied during evolution and detection as well as DQ excitation and recoupling was applied. During SPC5 recoupling, CW proton decoupling was employed, during evolution and detection SPINAL-64 decoupling.^62^

### Conformational Quantification

The peaks in the proteins’ DQ/SQ spectra, recorded at 800 MHz spectrometer, were assigned to each conformation according to their respective chemical shift. Each peak was volume integrated using a rectangular box integration (Topspin 4.0.9). Rotamer distributions in percentages (given in Tables 2 and 4 and Tables S3 and S7) were determined as follows.

For the g_m_,g_m_ conformation, the volume of the rectangular boxes around respective Cα/Cβ and Cγ1/Cδ1 cross peaks (marked in yellow in Figure S7), was integrated and then divided by the full peak volume for the same side chain carbon (marked in blue in Figure S7) in order to get the population percentages (%). From the different g_m_,g_m_ population percentages determined for each side chain carbon peak an average value was calculated.

For an estimate of the helical population (also called index) we have integrated a rectangular box around the typical helical tail visible on the Cα/Cβ cross peak in the range 63–69 ppm (marked in black in Figure S7). The integrated volume was then compared to the full Cα peak integral in order to gain a measure for the relative population % shown in the tables. The detailed integrated areas used for each protein system are provided in Table S6.

Cα/Cβ and Cγ1/Cδ1 cross peaks for g_m_,g_m_ conformations are clearly separated from the other cross peaks also in unfolded proteins and thus can easily be quantified. In contrast, the selection of the α-helical part of a heterogeneously broadened continuum of overlapping chemical shifts can only give an estimate for a general trend, but should not be regarded as a quantification of the backbone conformational ensemble.

The fwhm was also measured for every peak visible in the DQ/SQ spectrum, and in Table S4 all the values are shown. Those values were then averaged to the fwhm average value, which was used for the discussion.

## Supporting Information Available

Detailed experimental NMR acquisition and processing parameters and additional spectra (PDF)
NMR raw data are available under https://bmrbig.org/released/bmrbig114
