# Supplementary material for: Isoleucine Side Chains as Reporters of Conformational Freedom in Protein Folding Studied by DNP-Enhanced NMR
Source: J Am Chem Soc. 2025 Apr 26;147(18):15867–79. doi: 10.1021/jacs.5c04159 (PMC12063180; doi:10.1021/jacs.5c04159)
Supplement: Supplementary file 1 — ja5c04159_si_001.pdf [file ja5c04159_si_001.pdf]

Supporting Information

Isoleucine Side Chains as Reporters of Conformational Freedom in Protein Folding Studied by DNP-Enhanced NMR

Leonardo Levorin[1,2], Nina Becker[1,2], Boran Uluca-Yazgi[1,2], Luis Gardon[1,2], Mirko Kraus[1,2], Marc Sevenich[1,2], Athina Apostolidis[1], Kai Schmitz[1,2], Neomi Rüter[1], Irina Apanasenko[1,2], Dieter Willbold[1,2], Wolfgang Hoyer[1,2], Philipp Neudecker[1,2], Lothar Gremer[1,2], Henrike Heise[1,2*]

[1]Institute of Physical Biology,
Heinrich-Heine-Universität Düsseldorf
40225 Düsseldorf, Germany

[2]Institute of Biological Information Processing (IBI-7: Structural Biochemistry)
Forschungszentrum Jülich
52425 Jülich, Germany

* Email: h.heise@fz-juelich.de

NMR RAW DATA

NMR raw data are available under https://bmrbig.org/released/bmrbig114

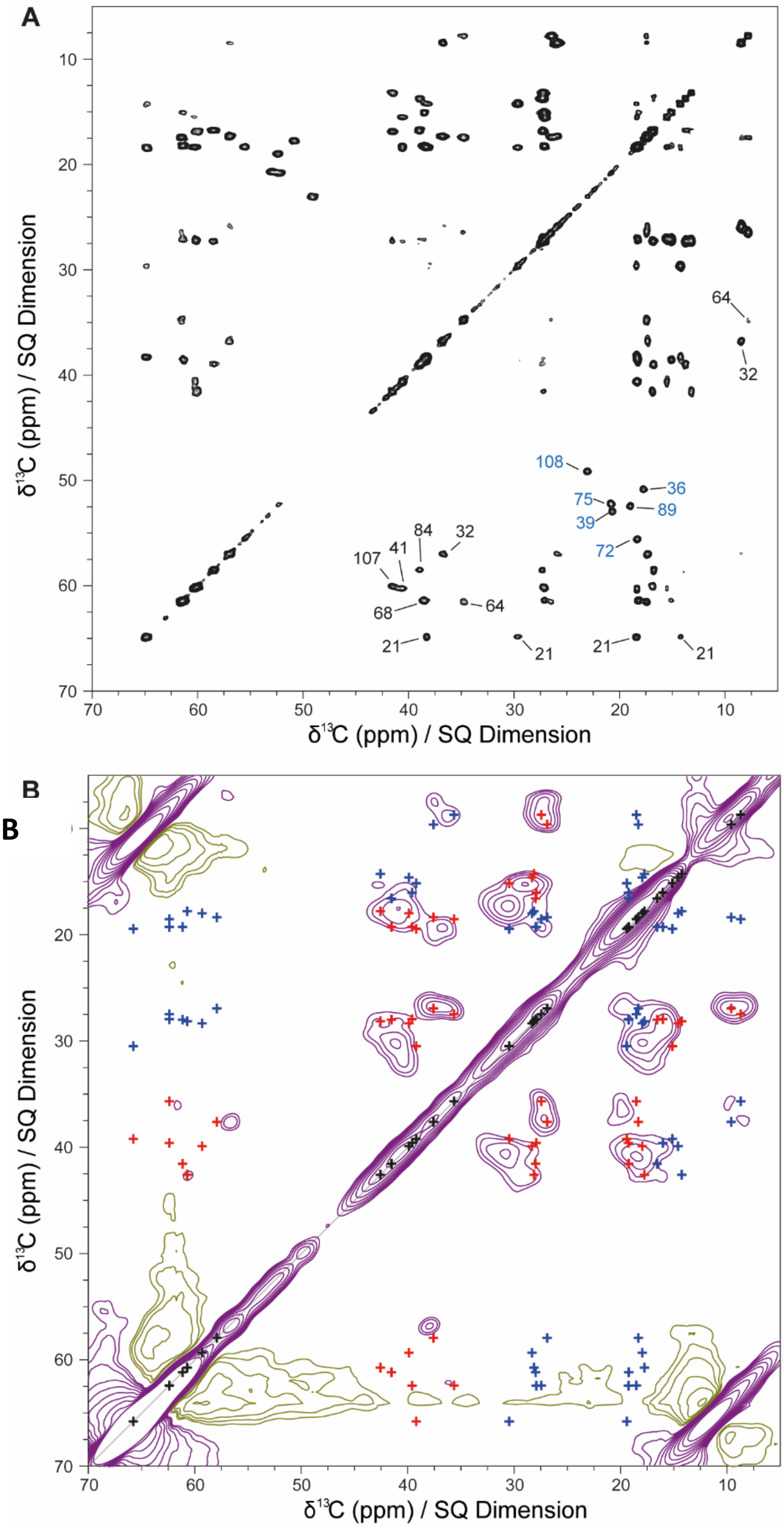

Figure S1: *Aliphatic 2D ^{13}C-^{13}C-TOCSY spectrum of [^{13}C,^{15}N]-Ala/Ile labeled GABARAP in solution at 25.0°C recorded with 13.6 ms FLOPSY-16 mixing at 600 MHz. Selected TOCSY cross peaks for Ile (black) and Ala (blue) are labeled according to their residue number. B) 2D PDSD of [^{13}C,^{15}N]-Ala/Ile labeled GABARAP in frozen solution (see Table S9) at a temperature of 100 K recorded at 800 MHz with a spinning speed of 11 kHz. The spectrum was processed with an unshifted squared cosine function. Positive contour lines are displayed in purple, negative in green. Simulated one-bond and two-bond cross peaks from solution NMR chemical shifts are indicated as red and blue crosses, respectively (Cα/Cβ cross peaks are affected by baseline perturbations from the strong glycerol diagonal signal).*

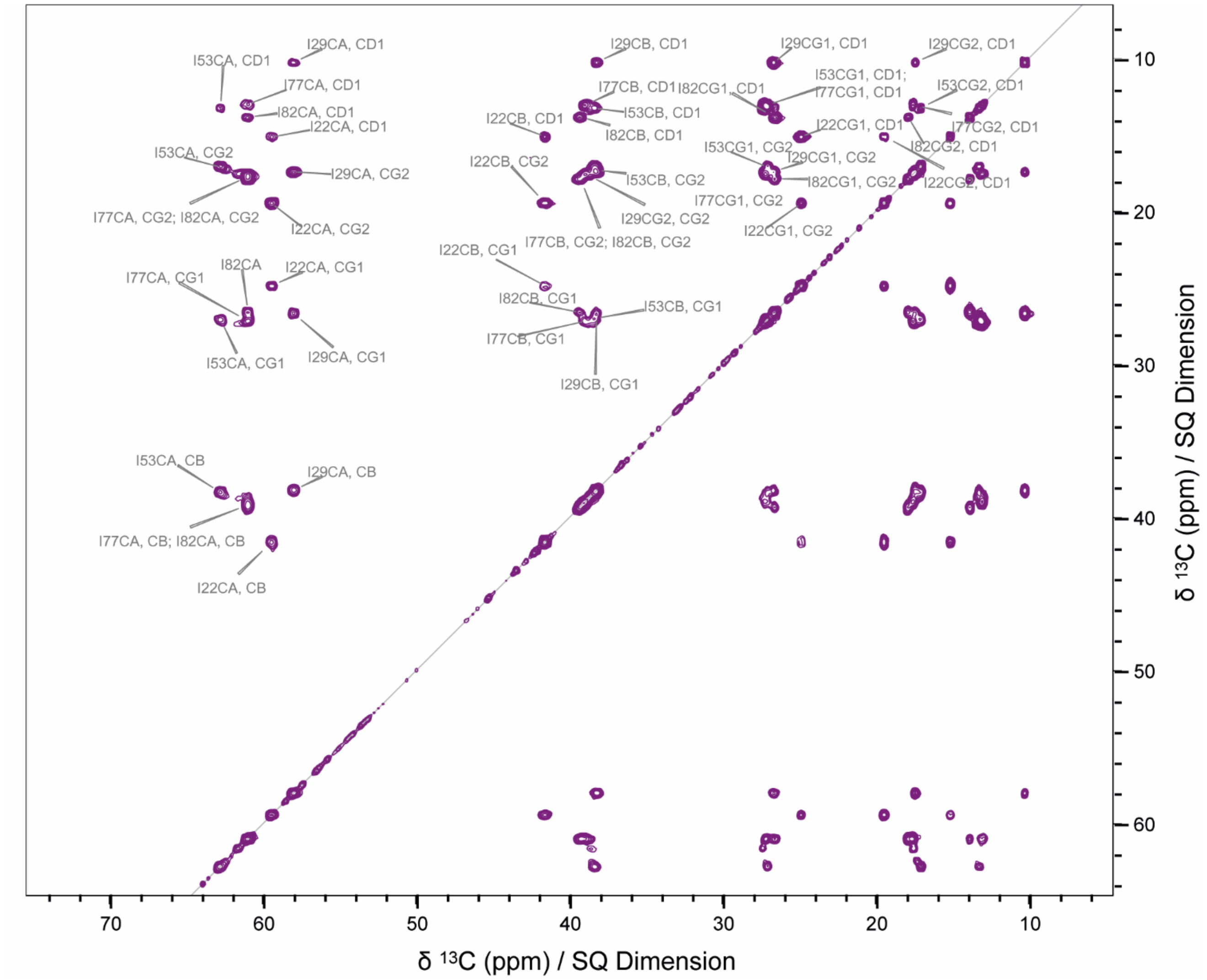

Figure S2: *Aliphatic 2D ^{13}C-^{13}C-TOCSY spectrum of [^{13}C,^{15}N]-Ile labeled PI3K SH3 at pH 6.8 in solution at 25.0°C. Sequence-specific cross peak assignments of all isoleucine side chains are indicated.*

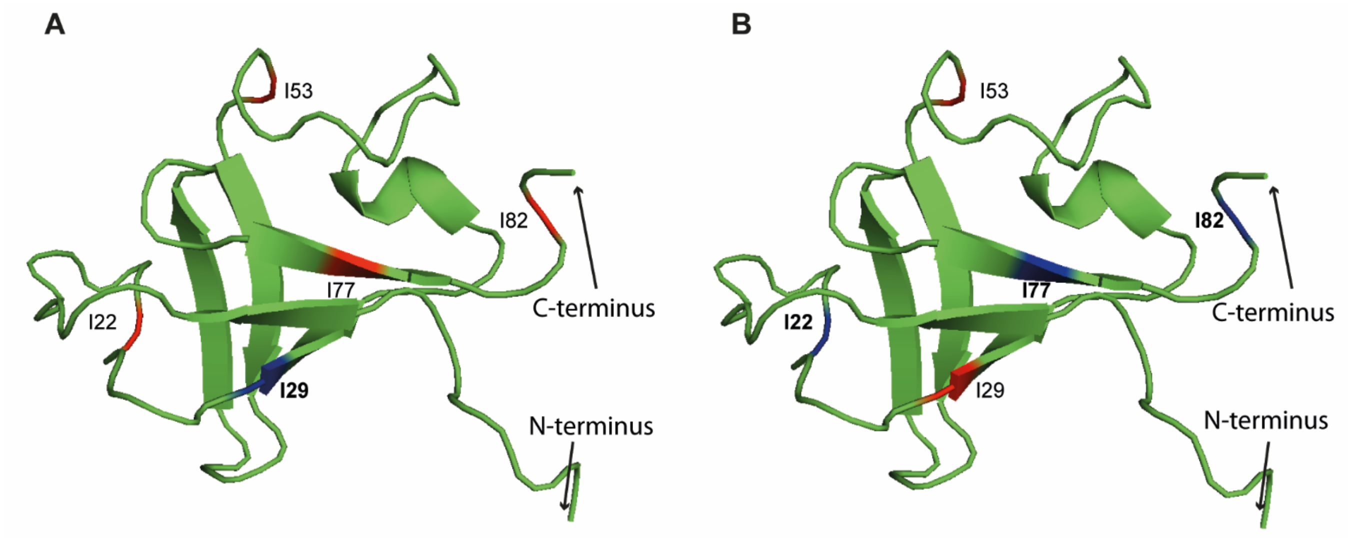
A
I53
I82
I77
C-terminus
I22
I29
N-terminus
B
I53
I82
I77
C-terminus
I22
I29
N-terminus

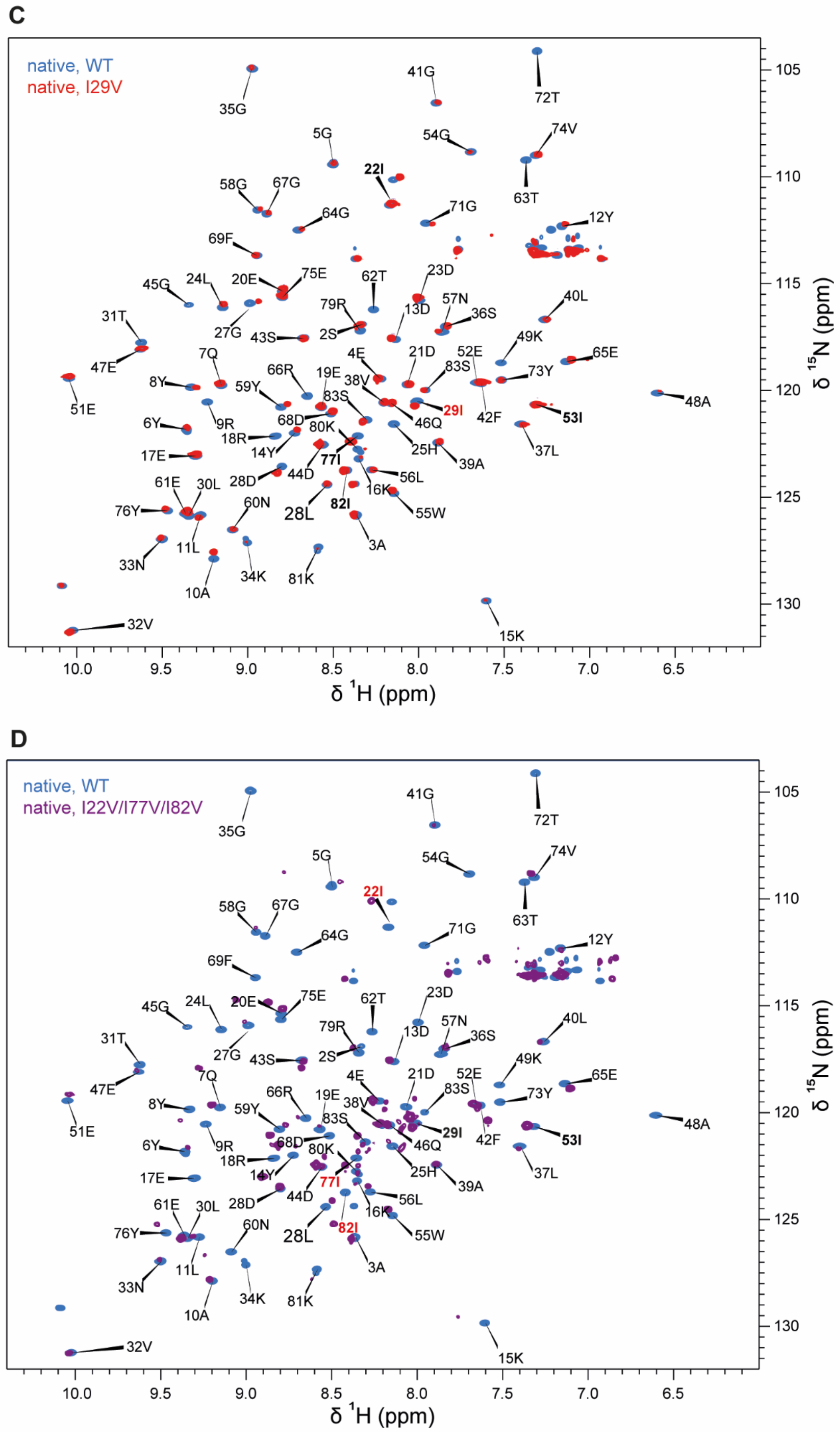

Figure S3: *I29V (A) and I22V/I77V/I82V (B) mutants with the respective substitution marked in blue in the 3D structure of PI3K SH3 in its native form (1PNJ). (C-D) Comparison of their respective HSQC spectra with the one recorded for the wildtype protein in the native form.*

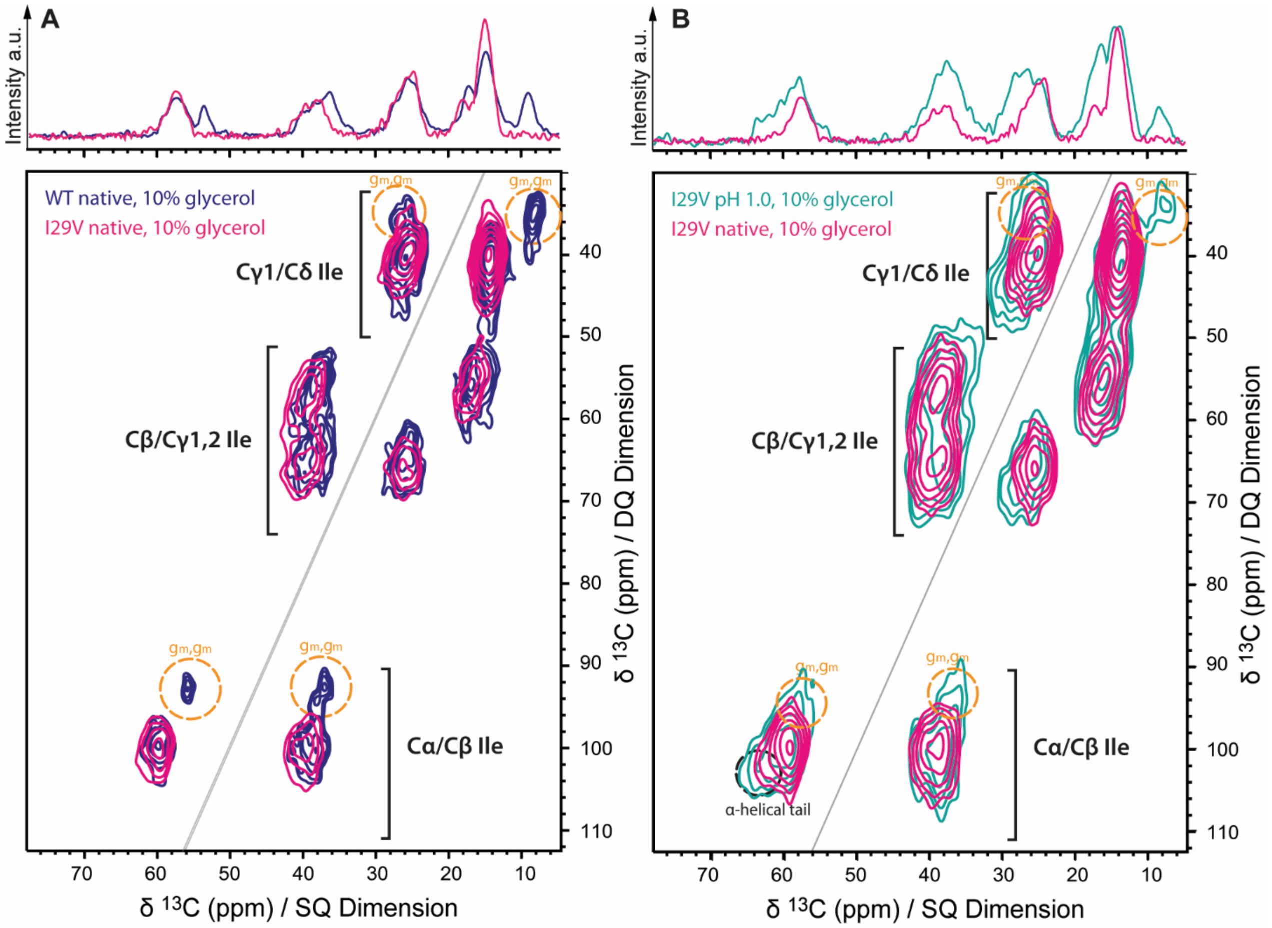

Figure S4: *2D ^{13}C-^{13}C correlation DQ/SQ spectra of Ile-labeled PI3K SH3 I29V variant in its native form recorded at cryogenic temperatures in frozen solution with 10% glycerol (blue), overlaid with the wildtype Ile-labelled PI3K SH3 spectra recorded in the same condition (pink) (A) and with the unfolded I29V variant spectra recorded at pH 1.0, 10% glycerol (cyan) (B). Spectra were processed with squared cosine functions in both dimensions using effective maximum acquisition times of 5.6 ms in the direct dimension and 2.9 ms (both spectra overlaid in A) or 1.4 ms (both spectra overlaid in B), respectively, in the indirect dimensions.*

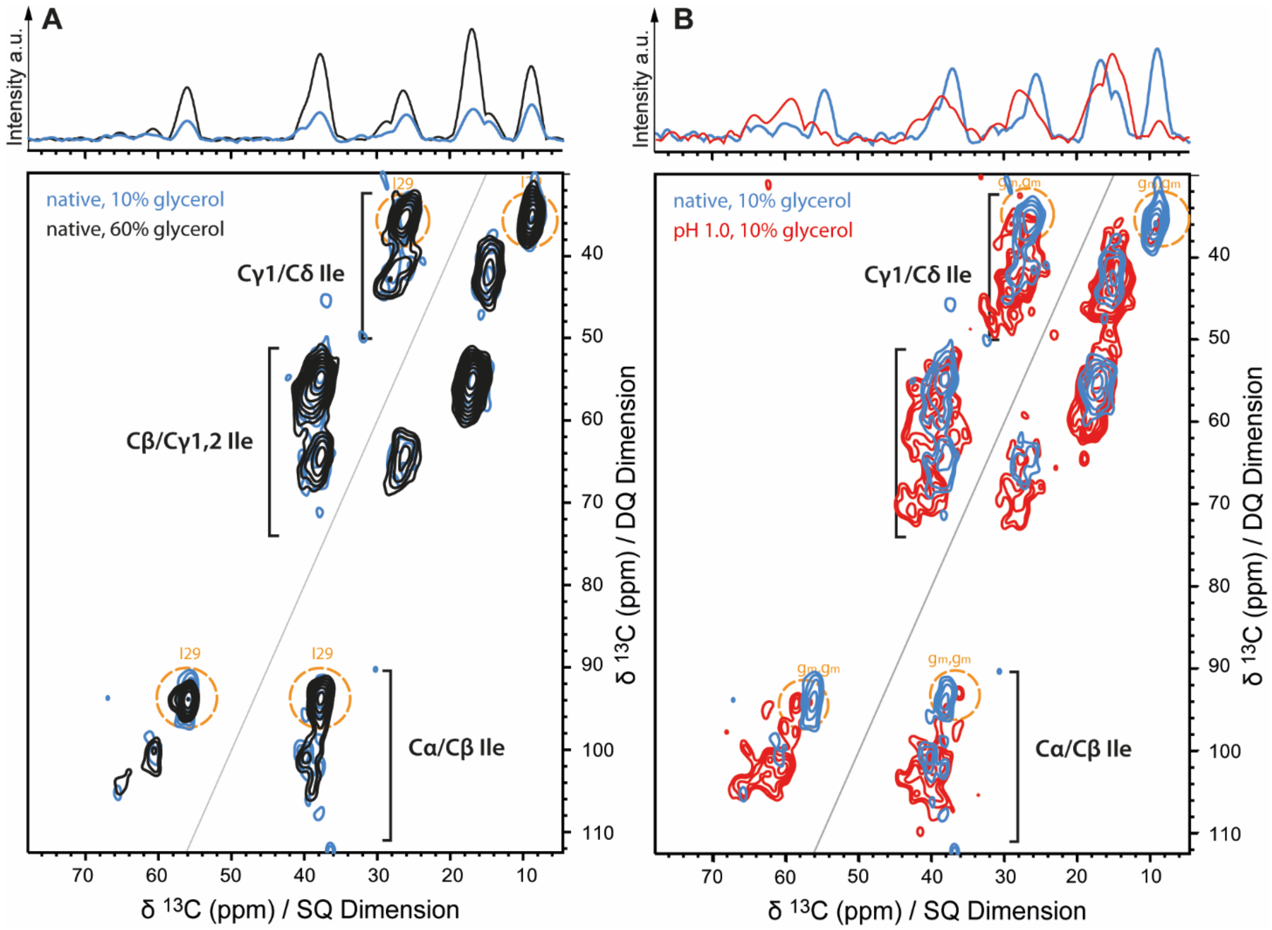

Figure S5: *2D ^{13}C-^{13}C correlation DQ/SQ spectra of Ile-labeled PI3K SH3 I22V/I77V/I82V variant in its native form recorded at cryogenic temperatures in frozen solution with 60% (black) and 10% glycerol (cyan) (A). Comparison of the same isoleucine-labeled PI3K SH3 variant in its native form in 10% glycerol (blue) with its unfolded state at pH 1.0 (red) (B).*

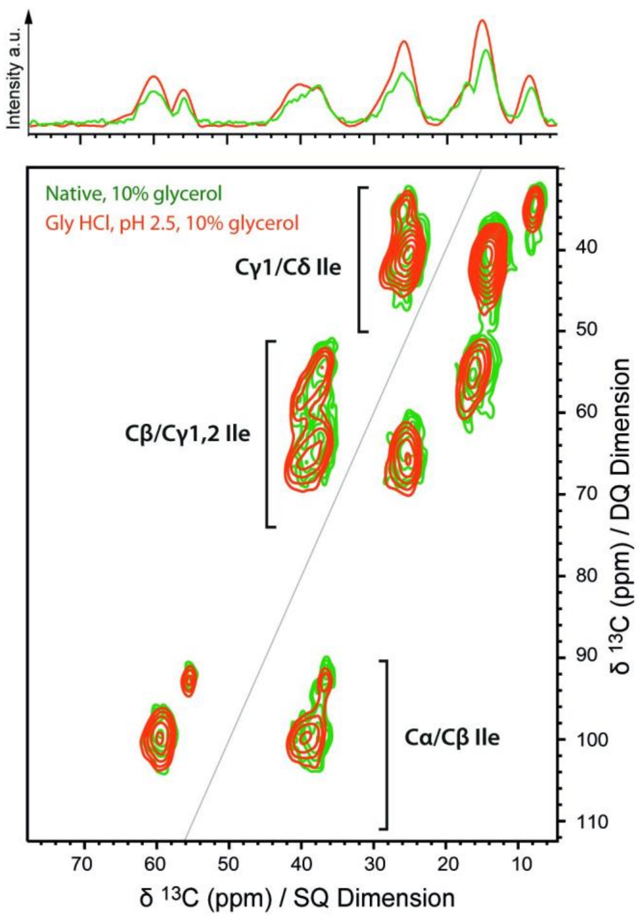

Figure S6: *2D ^{13}C-^{13}C correlation DQ/SQ spectra of isoleucine-labeled PI3K SH3 in its native form recorded at cryogenic temperatures in frozen solution with 10% glycerol (green) and in Gly HCl, pH 2.5 with 10% glycerol (orange).*

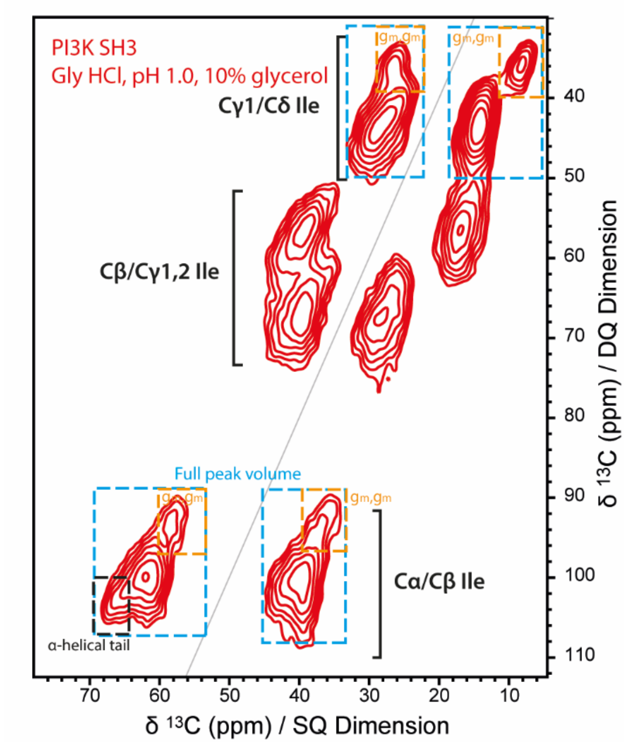

Figure S7: *2D ^{13}C-^{13}C correlation DQ/SQ spectra of isoleucine labeled PI3K SH3 in its unfolded state in 25mM glycine HCl, pH 1.0. The areas for the volume integration are highlighted in yellow (g_m,g_m state), black (α-helical), and blue (full peak).*

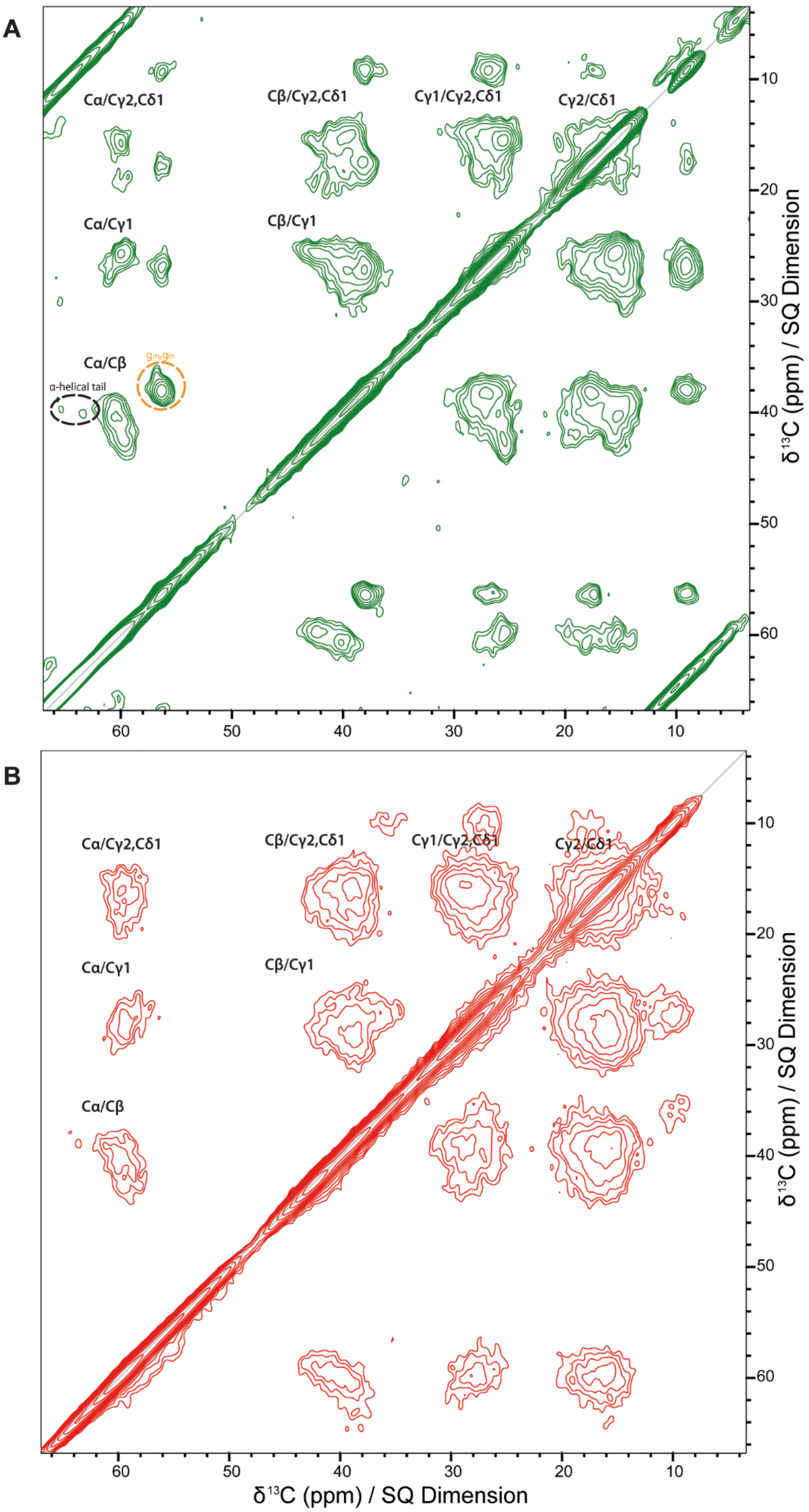

A
Cα/Cγ2,Cδ1
Cβ/Cγ2,Cδ1
Cγ1/Cγ2,Cδ1
Cγ2/Cδ1
Cα/Cγ1
Cβ/Cγ1
Cα/Cβ
α-helical tail
δ 13C (ppm) / SQ Dimension
B
Cα/Cγ2,Cδ1
Cβ/Cγ2,Cδ1
Cγ1/Cγ2,Cδ1
Cγ2/Cδ1
Cα/Cγ1
Cβ/Cγ1
Cα/Cβ
δ 13C (ppm) / SQ Dimension
δ 13C (ppm) / SQ Dimension

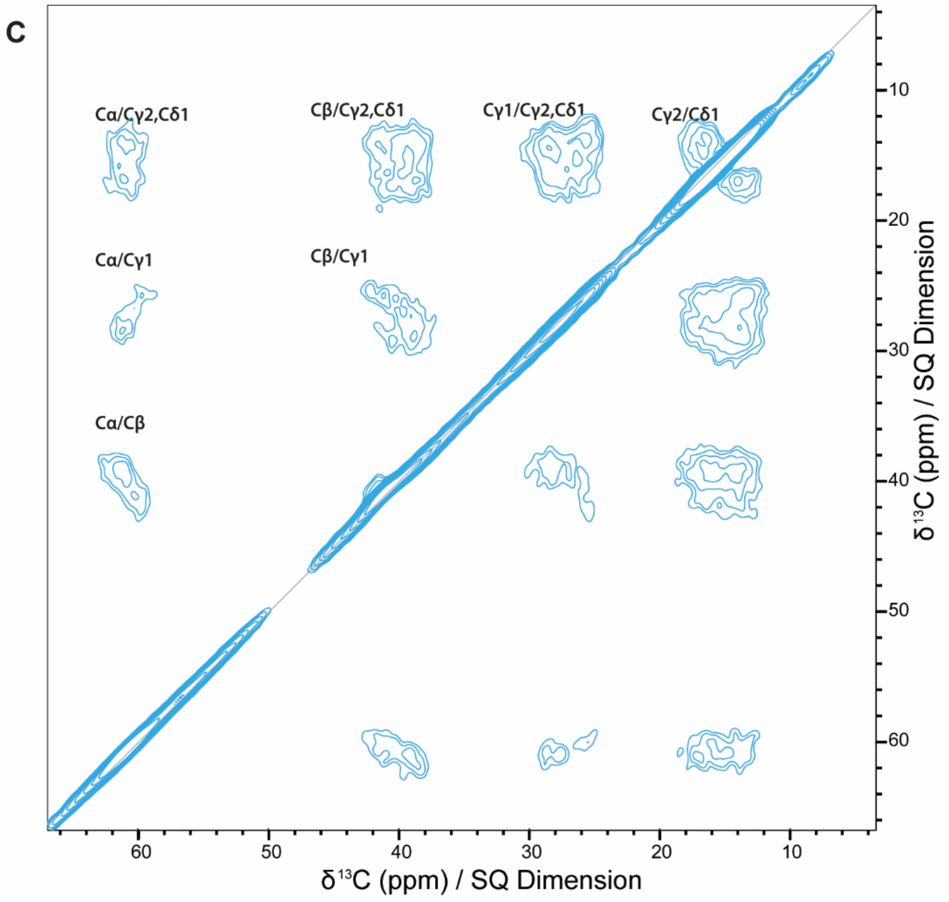

Figure S8: *PDSD spectra of PI3K-SH3 in its native form at pH 6.8 with 10% glycerol (A), in 25 mM Gly HCl, pH 1.0 with 10% glycerol (B) and in GdmCl, pH 2.5 with 10% glycerol (C). The areas of the* g_m,g_m *and α-helical signals for the Cα/Cβ crosspeak are marked with circles. PDSD mixing times were 20 ms (A) or 50 ms (B and C). Spectra were processed with squared sine bell functions with sine bell shifts of 3 in each dimension. (Contour levels are displayed at positive multipliers of 1.41).*

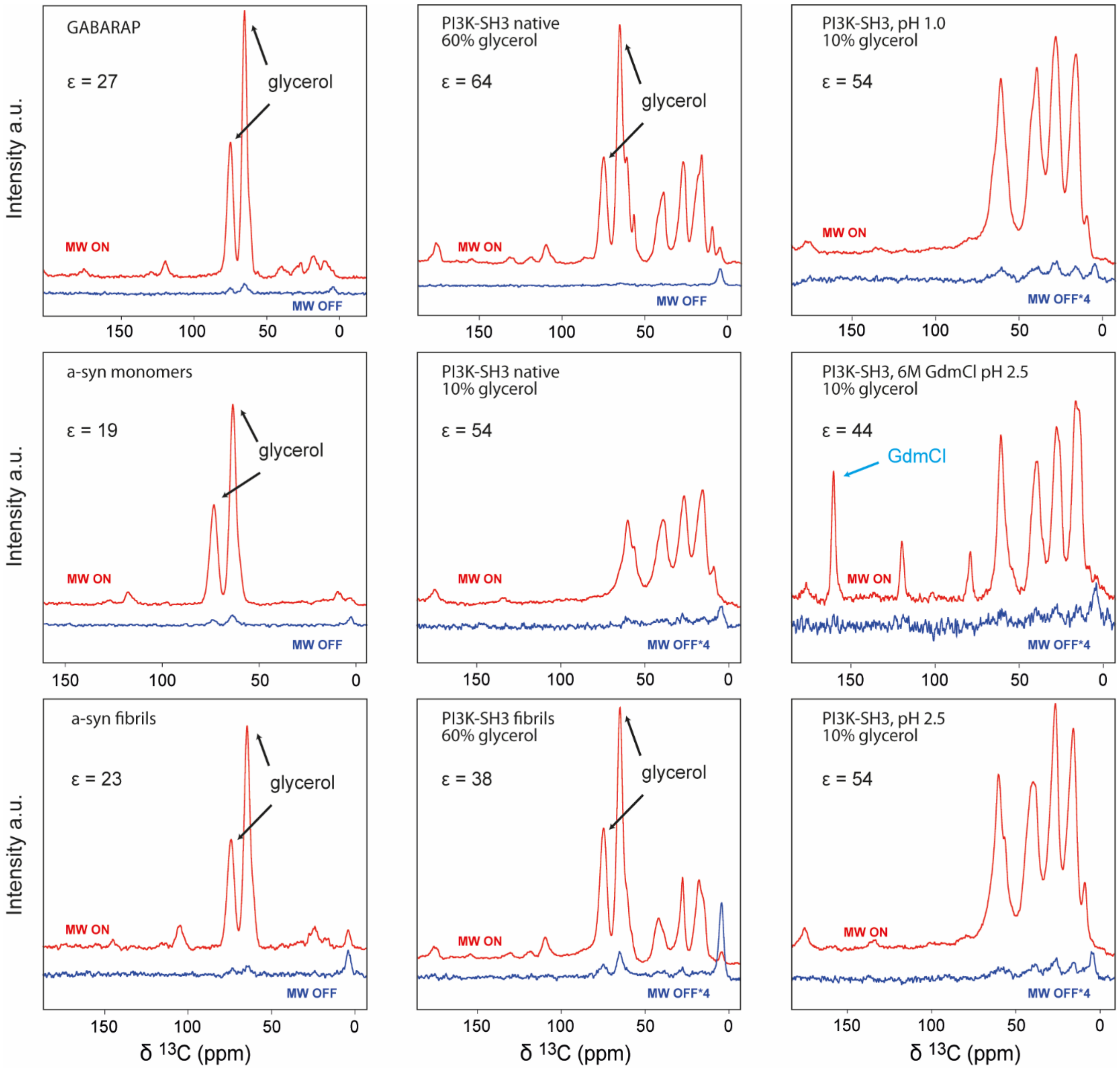

Figure S9: *^{13}C ssNMR spectra of all the samples recorded with and without microwave irradiation, with the respective DNP enhancement (ε) indicated.*

Table S1: *line widths of signals in the spc5 spectrum of GABARAP in ppm.*

Cα/Cβ	Cα/Cβ (I32 g_m,g_m)	Cα/Cβ (I64 g_m,g_m)	Cβ/Cα	Cβ/Cα (I32 g_m,g_m)	Cβ/Cα (I64 g_m,g_m)	Cγ/Cδ	Cδ/Cγ	Cδ/Cγ (g_m,g_m)	CG(g_m,g_m)
2.8	1.3	1.4	3.6	1.4	1.6	2.3	1.7	1.7	2.0

Table S2: *Populations of different conformations measured in GABARAP spc5 spectrum.*

I32 (g_m,g^m)	I64 (g_m,g_m)	I21 total	I21 (helical)	I21 (coil)
16±2%	16±2%	15±2%	8±2%	7±2%

Table S3: *Populations of different conformations measured in AIN, a-syn and PI3K SH3 spc5 spectra.*

Sample	Cα/Cβ (g_m,g_m)	Cβ/Cα (g_m,g_m)	Cγ/Cδ (g_m,g_m)	Cδ/Cγ (g_m,g_m)	Average value (g_m,g_m) (%)	Index for Cα/Cβ α-helical population (%)
AIN	15±1	14±1	14±1	11±1	14±1	15±1
α-syn monomers	14±2	17±2	14±2	15±2	15±2	18±2
a-syn fibrils	24±6	11±6	17±6	14±6	17±6	9±6
PI3K SH3 native, pH 6.8, 60% glycerol	26±1	23±1	26±1	26±1	25±1	4±1
PI3K SH3 native, pH 6.8, 10% glycerol	23±1	22±1	23±1	23±1	23±1	1±1
PI3K SH3, pH 2.5, 10% glycerol	20±1	21±1	20±1	19±1	20±1	7±1
PI3K SH3, 6M GdmCl, pH 2.5, 10% glycerol	8±1	7±1	9±1	9±1	8±1	0±1
PI3K SH3, pH 1.0, 10% glycerol	16±1	16±1	14±1	19±1	16±1	16±1
PI3K SH3 fibrils	11±3	11±3	9±3	11±3	11±3	6±3

Table S4: *line widths of signals in spc5 spectra of AIN, a-syn and PI3K SH3 in ppm.*

Sample	Cα/Cβ (g_m,g_m)	Cα/Cβ	Cβ/Cα	Cβ/Cα (g_m,g_m)	Cγ/Cδ	Cγ/Cδ (g_m,g_m)	Cδ/Cγ	Cδ/Cγ (g_m,g_m)
AIN	2.7	2.9	2.6	2.3	3.2	2.2	2.2	2. 6
α-syn fibrils	2.0	3.5	2.6	1.7	1.7	1.6	2.1	1.8
α-syn monomers	4.9	3.3	4.9	4.1	2.7	2.7	3.6	3.1
PI3K SH3 native, pH 6.8, 60% glycerol	2.8	3.6	3.2	3.1	2.5	2.4	2.2	2.0
PI3K SH3 native, pH 6.8, 10% glycerol	4.6	1.7	4.4	2.0	4.0	4.0	2.8	2.3
PI3K SH3, pH 2.5, 10% glycerol	4.0	5.5	5.8	4.0	3.9	4.2	3.3	3.4
PI3K SH3, 6 M GdmCl pH 2.5, 10% glycerol	4.0	4.4	5.2	5.2	3.9	3.1	4.0	3.0
PI3K SH3, pH 1.0, 10% glycerol	6.0	5.4	4.2	4.7	3.5	3.2	3.0	2.8
PI3K SH3 fibrils, pH 2.5, 60 % glycerol	4.2	4.3	2.5	2.1	3.3	2.5	2.1	2.5

Table S5: *Population values for each conformation detected for I29 and I53 in the isoleucine-labeled PI3K SH3 I22V/I77V/I82V variant, from the spectrum shown in Fig. S6*

<table>
<tr><td>60% glycerol</td><td>g_m,g_m (%)</td><td>Helical (%)</td><td>Coil (%)</td></tr>
<tr><td>Cα/Cβ</td><td>53±1</td><td>19±1</td><td>28±1</td></tr>
<tr><td>Cβ/Cα</td><td>50±1</td><td>22±1</td><td>28±1</td></tr>
<tr><td>Cγ/Cδ</td><td>54±1</td><td colspan="2">46±1</td></tr>
<tr><td>Cδ/Cγ</td><td>50±1</td><td colspan="2">50±1</td></tr>
<tr><td colspan="4"></td></tr>
<tr><td>10% glycerol</td><td>g_m,g_m (%)</td><td>Helical (%)</td><td>Coil (%)</td></tr>
<tr><td>Cα/Cβ</td><td>48±1</td><td>14±1</td><td>38±1</td></tr>
<tr><td>Cβ/Cα</td><td>46±1</td><td>16±1</td><td>38±1</td></tr>
<tr><td>Cγ/Cδ</td><td>46±1</td><td colspan="2">54±1</td></tr>
<tr><td>Cδ/Cγ</td><td>46±1</td><td colspan="2">54±1</td></tr>
<tr><td colspan="4"></td></tr>
<tr><td>pH 1.0</td><td>g_m,g_m (%)</td><td>Helical (%)</td><td>Coil (%)</td></tr>
<tr><td>Cα/Cβ</td><td>29±2</td><td>23±2</td><td>48±2</td></tr>
<tr><td>Cβ/Cα</td><td>26±2</td><td>24±2</td><td>50±2</td></tr>
<tr><td>Cγ/Cδ</td><td>34±2</td><td colspan="2">66±2</td></tr>
<tr><td>Cδ/Cγ</td><td>33±2</td><td colspan="2">67±2</td></tr>
</table>

Table S6: *Detailed integrated areas of each peak for α-syn, AIN and PI3K SH3.*

	Cα/Cβ			Cβ/Cα		Cγ/Cδ		Cδ/Cγ	
	g_m,g_m	helical	total	g_m,g_m	total	g_m,g_m	total	g_m,g_m	total
α-syn	F2: 60.0-55.0 ppm<br>F1: 88.0-97.5 ppm	F2: 69.2-64.9 ppm<br>F1: 103.1-108.9 ppm	F2: 69.2-55.0 ppm<br>F1: 88.0-108.9 ppm	F2: 38.4-33.1 ppm<br>F1: 89.1-97.3 ppm	F2: 46.1-33.1 ppm<br>F1: 89.1-111.2 ppm	F2: 29.7-25.2 ppm<br>F1: 33.5-39.6 ppm	F2: 33.7-23.2 ppm<br>F1: 33.5-50.2 ppm	F2: 11.8-6.17 ppm<br>F1: 30.5-40.0 ppm	F2: 20.0-6.17 ppm<br>F1: 30.5-50.3 ppm
AIN	F2: 60.7-55.3 ppm<br>F1: 88.0-97.5 ppm	F2: 68.0-64.2 ppm<br>F1: 98.5-109.6 ppm	F2: 68.0-55.3 ppm<br>F1: 88.0-109.6 ppm	F2: 39.8-33.3 ppm<br>F1: 87.8-96.8 ppm	F2: 46.2-33.3 ppm<br>F1: 87.8-114.1 ppm	F2: 29.5-24.4 ppm<br>F1: 30.5-36.8 ppm	F2: 33.6-21.5 ppm<br>F1: 30.5-51.1 ppm	F2: 11.5-6.5 ppm<br>F1: 30.0-41.5 ppm	F2: 19.5-6.5 ppm<br>F1: 30.0-49.1 ppm
PI3K SH3	F2: 59.0-53.5 ppm<br>F1: 88.0-97.5 ppm	F2: 69.0-64.3 ppm<br>F1: 97.5-108.5 ppm	F2: 69.0-53.5 ppm<br>F1: 88.0-108.5 ppm	F2: 40.0-34.0 ppm<br>F1: 87.5-96.8 ppm	F2: 45.5-34.0 ppm<br>F1: 87.5-111.1 ppm	F2: 29.5-23.5 ppm<br>F1: 30.3-37.5 ppm	F2: 33.5-23.5 ppm<br>F1: 30.3-48.3 ppm	F2: 11.8-6.0 ppm<br>F1: 28.0-40.0 ppm	F2: 18.8-6.0 ppm<br>F1: 28.0-49.0 ppm

Table S7: *Populations of different conformations measured in PI3K SH3 PDSD spectrum.*

Sample	Cα/Cβ (g_m,g_m) (%)	Cβ/Cα (g_m,g_m) (%)	Average value (g_m,g_m) (%)	Index for Cα/Cβ α-helical population (%)
PI3K SH3 native, pH 6.8, 60% glycerol	27±2	28±2	28±2	8±2
PI3K SH3, 6M GdmCl pH 2.5, 10% glycerol	9±1	7±1	8±1	4±1
PI3K SH3, pH 1.0, 10% glycerol	15±2	13±2	14±2	18±2

Table S8: *Experimental details for spc5 spectra. SPC5 double quantum excitation and reconversion times were 488 µs. Full data available here: https://bmrbig.org/released/bmrbig114*

#	sample	Buffer	Polarizing agent	# t1 incr.	t_1 max (ms)	number of scans per increment	total exp. time (h)	Enhancement factor ε
**1**	GABARAP	60/30/10 d_8 glycerol/D_2O/H_2O	2.5 mM Amupol	394	6	48	36.93	27
**2**	AIN	60/30/10 d_8 glycerol/D_2O/H_2O	2.5 mM Amupol	316	6	32	19.75	64
**3**	α-syn monomers	60/30/10 d_8 glycerol/D_2O/H_2O	2.5 mM Amupol	100	1.5	240	46.85	19
**4**	α-syn fibrils	60/30/10 d_8 glycerol/D2O/H2O	2.5 mM Amupol	200	3	112	43.73	23
**5**	PI3K SH3	60/30/10 d_8, glycerol/ D_2O/25 mM Na_2HPO_4/NaH_2PO_4, pH 6.8,	5 mM M-Tinypol	200	3	112	43,73	64
**6**		10/80/10 d_8, glycerol/ D_2O/25 mM Na_2HPO_4/NaH_2PO_4, pH 6.8,	5 mM M-Tinypol	200	3	112	43.73	54
**7**		50 mM glycine/HCl, pH 2.5, 10% d_8, $^{12}C_3$ glycerol	5 mM M-Tinypol	190	2.9	112	41.55	54
**8**		6M GdmCl, pH 2.5, 10% d_8, $^{12}C_3$ glycerol	5 mM M-Tinypol	190	2.9	112	41.55	44
**9**		50 mM glycine/HCl, pH 1.0, 10% d_8, $^{12}C_3$ glycerol	5 mM M-Tinypol	200	3	112	43.73	54
**10**	PI3K SH3 fibrils	60/30/10 d_8 glycerol/D_2O/H_2O	5 mM M-Tinypol	302	4.6	32	18.88	38
**11**	I29V PI3K SH3	10/80/10 d_8, glycerol/ D_2O/25 mM Na_2HPO_4/NaH_2PO_4, pH 6.8,	5 mM M-Tinypol	200	3	112	43.73	64
**12**		50 mM glycine/HCl, pH 1.0, 10% d_8, $^{12}C_3$ glycerol	5 mM M-Tinypol	93	1.4	112	20.3	44

**13**		60/30/10 d_8, glycerol/ D_2O/25 mM Na_2HPO_4/NaH_2PO_4, pH 6.8,	5 mM M-Tinypol	200	3	112	43.73	64
**14**	122V/I77V /I82V PI3K SH3	10/80/10 d_8, glycerol/ D_2O/25 mM Na_2HPO_4/NaH_2PO_4, pH 6.8,	5 mM M-Tinypol	200	3	112	43.73	64
**15**		50 mM glycine/HCl, pH 1.0, 10% d_8, $^{12}C_3$ glycerol	5 mM M-Tinypol	200	3	112	43.73	54

Table S9: *Experimental details for pdsd spectra. Full data available here: https://bmrbig.org/released/bmrbig114*

#	sample	Buffer	Polarizing agent	# t1 incr.	t_1 max (ms)	mixing time (ms)	number of scans	total exp. time (h)	Enhancement factor ε
100	GABARAP	60/30/10 d_8 glycerol/D_2O/H_2O	2.5 mM Amupol	576	6.5	10	64	51	27
101	PI3K SH3	60/30/10 d_8, glycerol/ D_2O/25 mM Na_2HPO_4/NaH_2PO_4, pH 6.8	5 mM M-Tinypol	220	5	20	16	4.92	64
102		50 mM glycine/HCl, pH 1.0, 10% d_8, $^{12}C_3$ glycerol	5 mM M-Tinypol	480	6.7	50	64	43.22	54
103		6M GdmCl, pH 2.5, 10% d_8, $^{12}C_3$ glycerol	5 mM M-Tinypol	480	6.7	50	64	43.22	44

Table S10: *Detailed processing parameters for spc5 spectra.*

#	sample	Window function	Sine bell shift SSB	AQeff SQ/DQ (msec)	Td_{eff}	Coutour level
1	GABARAP	-	-	4.5/2.1	400/140	1.31
2	AIN	QSINE	2/2	5.6/1.5	500/100	1.31
3	α-synuclein (monomeric)	QSINE	2/2	5.6/1.5	500/100	1.31
4	α-synuclein (fibrils)	QSINE	2/2	4.5/1.5	500/100	1.31
5	PI3K SH3	QSINE	2/2	5.6/2.9	500/190	1.31
6		QSINE	2/2	5.6/2.9	500/190	1.31
7		QSINE	2/2	5.6/2.9	500/190	1.31
8		QSINE	2/2	5.6/2.9	500/190	1.31
9		QSINE	2/2	5.6/2.9	500/190	1.31
10	PI3K SH3 fibrils	QSINE	2/2	5.6/2.4	500/160	1.31
11	I29V PI3K SH3	QSINE	2/2	5.6/2.9<br>5.6/1.4	500/190<br>500/93	1.31
12		QSINE	2/2	5.6/1.4	500/93	1.31
13	122V, I77V, I82V PI3K SH3	QSINE	2/2	5.6/2.9	500/190	1.31
14		QSINE	2/2	5.6/2.9	500/190	1.31
15		QSINE	2/2	5.6/2.9	500/190	1.31
